# Validation of a Spanish version of the health-related quality of life (HRQoL) measure for Chronic Otitis Media (COMQ-12)

**DOI:** 10.1186/s12955-020-01616-5

**Published:** 2020-11-10

**Authors:** Ana M. Otoya-Tono, Lucía C. Pérez-Herrera, Daniel Peñaranda, Sergio Moreno-López, Ricardo Sánchez-Pedraza, Juan Manuel García, John S. Phillips, Augusto Peñaranda

**Affiliations:** 1 Fundación Universitaria de Ciencias de la Salud – Hospital de San José, Bogotá, Colombia; 2 Universidad de Los Andes, Cra 1 Nº 18A – 12 Bogotá, Colombia; 3 Otolaryngology and Allergology Research Groups, Unimeq-Orl, Bogotá, Colombia; 4 Clinical Research Institute, Universidad Nacional de Colombia, Bogotá, Colombia; 5 Hospital Universitario Fundación Santa Fe de Bogotá, Cra. 7 #117 – 15, Bogotá, Colombia; 6 Ear, Nose and Throat Department, Norfolk and Norwich University Hospital Foundation Trust, Norwich, Norfolk, UK

**Keywords:** Chronic Suppurative Otitis Media, Health-related quality of life, COMQ-12, Validity

## Abstract

**Background:**

Evaluation of health-related quality of life (HRQoL) is considered an important aspect of clinical assessment and health research. Chronic Otitis Media (COM) is related to the quality of life deterioration subsequent to COM symptoms, social communication impairments, and lower work performance. However, there is no reliable information regarding the impact of this disease on health and quality of life in many resource-poor countries. Therefore, we translated into Spanish the Chronic Otitis Media Questionnaire-12 (COMQ-12) for the evaluation of HRQoL of Chronic Otitis Media (COM) in adult patients. Also, we assessed the psychometric properties of the Spanish version of the questionnaire.

**Methods:**

Two otology referral centers in Bogotá, Colombia were included. The Spanish version of COMQ-12 was applied twice to 200 adult patients with confirmed COM diagnosis and 31 healthy controls to perform the validation process and assess the internal consistency of this questionnaire. Psychometric characteristics (internal consistency, test–retest reliability, and construct validity) of the COMQ-12 were assessed. Exploratory Factor Analysis and Confirmatory Factor Analysis were conducted via structural equation modeling to test the questionnaire’s structure.

**Results:**

The Spanish version of the COMQ-12 showed good internal consistency (Cronbach’s Alpha: 0.86, McDonald’s Omega: 0.89). Coefficients corresponding to Lin’s Concordance test and test–retest reliability were 0.95 and 0.83 respectively. Correlation between the Visual Analogue Scale (VAS) and the COMQ-12 was 0.68 (95% CI 0.59–0.75, *p* value < 0.001). Factor analysis of the Spanish version of the COMQ-12 indicated a questionnaire structure with three domains: smelly discharge related symptoms; hearing loss related symptoms; and impact on work, lifestyle, and health services.

**Conclusion:**

This Spanish version of the COMQ-12 showed high reliability and high internal consistency. This questionnaire can be used as an objective clinical tool to assess the HRQoL of patients who have a COM diagnosis.

**Trial registration:**

Hospital Universitario Fundación Santa Fe, Ethical Committee Registration ID: CCEI-8807-2018. Hospital de San José, Ethical Committee: Record number 500, DI-I-0632-18.

## Background

Chronic inflammation of the middle ear and mastoids, or Chronic Otitis Media (COM), is a very common medical problem worldwide affecting about 2% of the population [1]. Its prevalence varies considerably between populations, but it is most common in low-income and middle-income countries [2]. Current advances in the treatment of COM have contributed to a global downward trend in its incidence and complication rates [3,4]. Nevertheless, in many resource-poor countries, the prevalence of COM remains relatively high due to the combination of poverty, a dearth of specialists, and the inadequacies of public health policies [5]. In Colombia, the Integrated System for the Social Protection (SISPRO) reports a prevalence of COM of 0.131% (20,777 reported cases in 2017) [6]. However, this prevalence is probably underestimated due to the outsourcing in the Colombian healthcare system, the limited access to health services in some areas of the country, and the lack of specialists [7,8]. Therefore, there is no reliable information regarding the prevalence of this condition and its impact on health and quality of life.

Indeed, COM is related to quality of life deterioration after recurrent smelly discharge, hearing loss, tinnitus, and balance abnormalities [9]. Hearing impairment caused by otitis media increases with age, with a prevalence of 9.34 per ten thousand in the first year of life and 45.05 per ten thousand for those aged 65 to 74 years [2]. Therefore, social communication difficulties and lower work performance are associated with COM symptoms [2]. Moreover, its impact on patient quality of life is over 2 million DALYs (Disability-Adjusted Life Years) [10].

Evaluation of health-related quality of life (HRQoL) is considered an important aspect of clinical assessment and health research [^11^]. Measurement of HRQoL, particularly after treatment, has become an important feature to guide therapeutic decision making in clinical practice [^12^]. There are some HRQoL instruments for COM such as COMOT-15 [13], CES [14], COMBI [15], ZCMEI-21 [16], and recently, the COMQ-12 questionnaire [1]. ZCMEI-21 questionnaires have been translated to different languages such as: English [17], Japanese [18], Italian [19] and Chinese [20]. Likewise, COMQ-12 questionnaire has been translated and validated to different languages including: Indian Kannada [12], Dutch [21], Serbian [9], Portuguese [22], Russian [23], Korean [24], Turkish [25], and Italian [26]. However, there is no quality of life questionnaire for COM validated in Spanish language, and additional psychometric evaluation is required.

The original English version of the COMQ-12 was developed and validated by Phillips et al. This questionnaire is a clinically useful tool for assessing the quality of life in patients with COM [1]. Bearing in mind that the English version of COMQ-12 is not applicable for native Spanish speakers, it was necessary to develop and validate a Spanish version of COMQ-12. Therefore, the objective of this study is the validation of the Spanish Language version of the COMQ-12 quality of life instrument by comparing the scores of the patients and the control group, as well as the test re-test differences. The validation of this questionnaire in Spanish is essential to assess the quality of life impact of COM on native Spanish speakers, and thus, provide patients with better care.

## Methods

Firstly, we requested Doctor John Phillips’s authorization to start the validation process. The original version of the COMQ-12 includes 12 self-assessment questions grouped in four categories: questions 1 through 7 inquire about the severity of symptoms, questions 8 and 9 are related to the impact of the disease on work and lifestyle, questions 10 and 11 ask about the impact of COM on health services, and question 12 is a Visual Analogue Scale that measures the global quality of life (QoL). Each item is scored from 0 to 5 according to the level of discomfort of the patient [1].

### Translation

We attached to the recommendations of the European Organization for Research and Treatment of Cancer (EORTC) quality of life group to carry out the translation process of the questionnaire [27]. Two native Spanish-speaking official interpreters translated the original COMQ-12 from English to Spanish. Translators were instructed to avoid using challenging or technical words when building the questionnaire. The concordance between these translations was assessed and no discrepancies were found regarding the content of the questionnaire. Subsequently, two native English-speaking official interpreters translated it back to English. Both versions of the questionnaire were checked by two bilingual neuro-otologists to confirm that the questions were comprehensible and preserved the original meaning of the English version of the COMQ-12. Afterward, five members of the Otolaryngology team from the Hospital Universitario Fundación Santa Fe de Bogotá (two neuro-otologists, two otolaryngologists, and a bilingual otolaryngology resident) reviewed and unified both versions of the questionnaire into a final version of the questionnaire. Finally, the Spanish version of the COMQ-12 was sent back to the authors of the original English version in order to be approved.

### Pilot test

The language adaptation was performed through a pilot test applied in a 15-min interview of ten patients with COM diagnosis who visited the institutions included in the study. All the questions of the COMQ-12 were read to the patients to assess their understanding. The patients described their experience answering each question of the questionnaire, such as whether the concepts in the questionnaire were familiar, whether they struggled to choose between the response options, whether they were able to understand the questions quickly or after considerable effort. No additional adjustments were required for the Spanish version of the COMQ-12.

### Participants and data

A statistical sample size calculation was performed for each psychometric characteristic. Firstly, we considered previous recommendations suggesting that a sample size of 200 (n ≥ 200) is enough to perform a factor analysis [28–31]. Secondly, in order to assess the concurrent validity criterion a sample size of 132 subjects was calculated to achieve a 5% significance level, a power of 80%, and a population correlation of 90%, all under a null hypothesis correlation of 0.75 [32]. Moreover, to assess the internal consistency using the Cronbach's alpha coefficient, a total of 144 subjects was estimated to achieve a null hypothesis level of 0.15, an alternate hypothesis of 0.4 with a 5% significance level, and a power of 80% for a total of 12 items [33]. Likewise, to evaluate the test–retest reliability a minimum of 149 subjects was estimated to achieve a difference of 0.1 between the correlations, a significance level of 5%, a power of 80%, a two-tailed correlation under the null hypothesis of 0.7 and the alternative hypothesis of 0.8 [33]. Finally, to assess the sensitivity to change of the questionnaire, a minimum of 56 patients was estimated with an average difference of 10 points and a pooled standard deviation of 8 points to reach a 5% level of significance and a power of 90%. This last calculation was based on Lehmann’s methodology for Wilcoxon’s paired rank tests [34].

Regarding the sample selection method, a non-probabilistic, consecutive sampling of the subjects who met the eligibility criteria was conducted. For this study, we refer to the definition of COM as chronic inflammation of the middle ear and mastoid which persists over 6 weeks to 3 months despite medical treatment [10].

Two hundred patients who visited Hospital Universitario Fundación Santa Fe de Bogotá and Hospital de San José and were diagnosed with COM agreed to participate in this prospective study. They were enrolled in the study between August 2018 and August 2019. All the enrolled patients were adults (over 18 years old), native Spanish speakers, and had confirmed diagnosis of COM. Two otologists performed the otoscopic examination, and their audiometric testing results were recorded in the database of the study. All patients completed the Spanish COMQ-12 questionnaire. Patients who had previous ear surgery, cognitive deficits, psychiatric disorders, severe comorbidities (e.g. cancer, HIV, and severe chronic concomitant diseases) were excluded from the study. An estimated number of 50 patients who reported a history of previous ear surgery were excluded from the study. Two patients who had cognitive deficits were excluded as well. Likewise, patients who reported any acute or chronic condition that could limit their ability to participate in the study, and those who refused to sign the informed consent were not included in the study.

In addition, the Spanish questionnaire was administered to a normative reference volunteer group of thirty-one healthy adults. Hospital staff members without previous history of middle ear disease and with normal audiometric testing composed this comparable convenience sample. The calculation of this control group sample size was established considering statistical recommendations to test the questionnaire’s discriminating properties between COM patients and healthy adults [28].

Additionally, all patients completed a sociodemographic questionnaire. The information included in this questionnaire was: sex, age, socioeconomic status, educational level, bilateral air- and bone- conduction hearing thresholds of the pure-tone audiograms, questions related to the history of the disease, and otoscopic findings. The socioeconomic status levels were defined as low-income levels (I and II), middle-income levels (III and IV), and high-income levels (V and VI) based on the classification of the Colombian National Administrative Department of Statistics, which uses income data, residential characteristics and cadastral/property base information to establish this stratification [35].

Once the purpose of the study was explained, the participants signed the informed consent form and completed the Spanish version of the COMQ-12 and the sociodemographic questionnaire. Even though some previous validations suggest an autonomous completion of the questionnaire [9], there is no consensus on the most reliable way to complete the COMQ-12 questionnaire [15]. Therefore, a trained medical doctor and two otologists were available to provide minimum assistance only if the subjects explicitly asked for it through the questionnaire. All subjects were requested to retake the test 15 or 30 days after the first visit. The same trained medical doctor and otologists who administered the questionnaire gathered the collected data.

### Otoscopic findings

Two otologists and a general practitioner from both medical institutions included in the study performed the otoscopic examination (Welch-Allyn Otoscope). According to previous recommendations about otology research [22], the otoscopic findings were classified into 5 groups: active squamous epithelium (cholesteatoma), perforated eardrum with discharge, inactive squamous epithelium (retraction, atelectasis, epidermolysis), dry perforated eardrum, and healed COM (intact tympanic membrane, tympanosclerosis). To ease the statistical analysis, these groups were also classified into two main clusters according to the following disease activity:

- Active COM: Active squamous epithelium (Cholesteatoma), and perforated eardrum with discharge (Wet perforation).
- Inactive COM: Inactive squamous epithelium (retraction, atelectasis, epidermolysis), dry perforated eardrum, and healed COM (Neo-tympanum, intact tympanic membrane, tympanosclerosis).

### Statistical analysis

Statistical analysis was performed using Stata 16MP and R 3.6.1 software. To achieve the validation of a new instrument such as the Spanish version of COMQ-12, psychometric characteristics (internal consistency, reliability, and construct validity) were assessed. These characteristics were assessed as follows: the degree of correlation among the items of the questionnaire and among the items that set up each domain (internal validity); the degree of consistency and stability of the obtained scores, that is, the level of homogeneity in the obtained measurements (reliability); the degree of similarity in the questionnaire scores compared with a reference or standard (criterion validity); the content validity or the fact that the items of the questionnaire adequately represent the theoretical domain to be measured; the instrument repeatability defined as the similarity among the obtained scores in the questionnaire under the same conditions but in two different time points (intra-observer reliability); the fact that the questionnaire scores can be considered and used as a valid measurement of the evaluated phenomenon (construct validity); and the questionnaire's ability to discriminate between individuals with and without the assessed condition (discriminant validity) [9,36,37].

The Exploratory Factor Analysis (EFA) was performed following the “principal-factor” method: the possible matrix factorization was verified through Bartlett's Test of Sphericity and Kaiser–Meyer–Olkin (KMO) Test. The polychoric correlation matrix was found for the EFA. The extraction of the axes for the factor analysis was based on the choice of eigenvalues greater than 1 and parallel analyses were used to confirm how many factors to retain. If required, a rotation of the axes will be carried out to obtain greater representativeness in terms of factor loads (rotation promax, varimax or quartimax). Confirmatory Factor Analysis (CFA) were run via Structural Equation Modelling (SEM) to test the questionnaire’s structure. The statistical tests were used to determine and compare goodness of fit of the models: absolute fit indices including Chi-squared test, χ^2^/*df* ratio and standardized root mean square residual (SRMR), relative fit indices like the Tucker-Lewis index (TLI), and indices of fit like the root mean square error of approximation (RMSEA), Bentler’s Comparative Fit index (CFI), and the coefficient of determination (CD). Additionally, to verify the obtained results in the factor analysis the sample was randomly split into two halves. An EFA was performed using the first half, and a CFA using the second half.

Concurrent validity was assessed using Spearman’s rho coefficient to establish the correlation between COMQ-12 items and the VAS (Q12). Two weeks after the first visit, the questionnaire was administered again to all the participants enrolled in the study. The results obtained in this second assessment were used to evaluate test re-test reliability through Lin's Concordance correlation coefficient calculation. On the other hand, Cronbach’s alpha and McDonald’s Omega coefficients were used to assess the internal consistency of the questionnaire. A Cronbach’s alpha value between 0.7 and 0.9 was considered enough to achieve a proper level of internal consistency [36,38]. McDonald’s Omega regards factor loadings of the items, and different studies show that it is one of the best alternatives to estimate reliability [39,40]. Finally, a Wilcoxon test was performed to evaluate the discriminant validity of the questionnaire and to compare the different values obtained from patients with active disease, and inactive disease as well as the control adults.

Ethics committee approval was received for this study from the ethics committee of the Hospital Universitario Fundación Santa Fe (CCEI-8807-2018) and the Hospital de San José (act number 500, DI-I-0632-18).

## Results

### Baseline demographic characteristics

A total of 200 patients diagnosed with COM who met the eligibility criteria were included in the study: 105 (52.5%) women with a mean age of 41.83 years (SD: 15.26). Table 1 describes the demographic characteristics of the patients, the number of years with the disease, and the otoscopic findings discriminated by ear. Of the 200 patients enrolled in the study, 69.5% of them belong to lower-income levels (Strata 1 and 2) and only 9% had attained a professional degree.

**Table 1:** Baseline demographic and clinical characteristics of the COM participants

Characteristic	n	%
Sex, f/m^a^	105/95	52.5/47.5
Age (Years)^b^	41.83 (15.26)	40.81 (18.02–85.47)
Socioeconomic status		
Low-income level (Strata 1 and 2)	139	69.5
Medium income level (Strata 3 and 4)	58	29
High-income level (Strata 5 and 6)	3	1.5
Educational level		
Primary education (Complete)	33	16.5
Secondary education	104	52
Technical degree	34	17
University education	18	9
Postgraduate degree	11	5.5
Disease duration (Years)^a^	26.13 (17.06)	23 (1–63)
Bilateral otitis	86	43
Previous contralateral surgery	6	3
Otoscopic examination—right ear		
Active squamous epithelium (Cholesteatoma)	15	7.5
Inactive squamous epithelium (Retraction, atelectasis, epidermolysis)	16	8
Perforated eardrum with discharge (Wet perforation)	56	28
Dry perforated eardrum	39	19.5
Healed COM (Neo-tympanum, intact tympanic membrane, tympanosclerosis)	22	11
Normal otoscopic examination	52	26
Otoscopic examination—left ear		
Active squamous epithelium (Cholesteatoma)	19	9.5
Inactive squamous epithelium (Retraction, atelectasis, epidermolysis)	14	7
Perforated eardrum with discharge (Wet perforation)	51	25.5
Dry perforated eardrum	34	17
Healed COM (Neo-tympanum, intact tympanic membrane, tympanosclerosis)	20	10
Normal otoscopic examination	62	31

^a^f/m: female/male

^b^Values are expressed in Mean (SD) and Median (Range)

### Internal consistency

The overall consistency of the instrument was 0.86 for Cronbach's Alpha and 0.89 for McDonald's Omega. The values of the alpha coefficient, when each item was removed, varied between 0.83 and 0.86 for Cronbach’s Alpha whereas, for the Omega coefficient, they varied between 0.86 and 0.89. The removal of an item from the questionnaire reduces the level of overall consistency of the instrument (Table 2). Regarding McDonald's Omega coefficient, the score for the "disease severity" domain was 0.89.

**Table 2:** Cronbach's alpha and McDonald's Omega values

Item	Item-scale correlation	Cronbach’s alpha (One item removed)	McDonald’s omega (One item removed)
Q1	0.56	0.84	0.88
Q2	0.43	0.85	0.89
Q3	0.67	0.83	0.88
Q4	0.63	0.83	0.88
Q5	0.50	0.84	0.89
Q6	0.58	0.84	0.88
Q7	0.63	0.83	0.88
Q8	0.50	0.84	0.88
Q9	0.56	0.84	0.88
Q10	0.29	0.86	0.89
Q11	0.45	0.85	0.89
Q12	0.57	0.84	0.89

### Test–retest reliability

All patients were asked to answer the questionnaire twice: at the first visit and a second visit (15 or 30 days after the first visit). Values for each of the domains remained stable, with a difference not greater than one point. Table 3 shows the results of the test–retest reliability measurement for each of the domains of the questionnaire. As shown in the table, Lin’s correlation coefficients were calculated for each domain. These correlations ranged from 0.82 (95% CI 0.78–0.87) to 0.94 (95% CI 0.92–0.96).

**Table 3:** Lin's concordance correlation coefficients

COMQ-12 domains	Lin’s coefficient	95% CI	
Symptom severity	0.94	0.92	0.96
Impact on lifestyle and work	0.88	0.85	0.91
Impact on health services	0.82	0.78	0.87
VAS	1.00	–	–
Total	0.94	0.93	0.96

### Construct validity

Patients completed the questionnaire in a median period of 11 min, ranging from 8 to 15 min. To appraise construct validity, the 12 items of the questionnaire and the hypothetical domains were considered in building the correlation matrix. Bartlett´s sphericity test (χ^2^ 849.92; (*p* < 0.001) showed that the correlation matrix was statistically different from the identity matrix, and these findings suggest a correlation between the items of the questionnaire. Also, the KMO measurement of sampling adequacy (0.86) showed that data was suitable for Factor Analysis. The correlated matrix factorization was verified, and the polychoric correlation matrix of the items was obtained. Considering the eigenvalues’ criterion, three factors were evaluated (eigenvalues of 4.53, 0.80, and 0.58), and a cumulative variance of 53.88% was obtained. Hence, an oblique (promax) rotation was performed.

All factor loadings are higher than 0.3 for any hypothetical domain. Thus, the item’s distribution and the domain framework would change significantly in contrast with the originally proposed structure. Correlations of 0.52 (factor 1 “smelly discharge related symptoms” and factor 2 “hearing-related symptoms”), 0.54 (factor 1 “smelly discharge related symptoms” and factor 3 “impact on lifestyle, work and health”), and 0.45 (factor 2 “hearing-related symptoms” and 3 “impact on lifestyle, work and health”) were found. A residuals value of 0.03% with absolute values superior to 0.05 was obtained. Considering that item Q12 is a VAS that measures the patient´s quality of life, there is a possibility that this item can load any factor of the questionnaire; therefore, this item was not included in the factor analysis. Table 4 shows the rotated factor matrix obtained.

**Table 4:** Rotated factor matrix

Item	Factor 1	Factor 2	Factor 3	Uniqueness
Q1	0.08	0.10	0.71	0.34
Q2	0.04	− 0.02	0.71	0.47
Q3	0.85	0.02	0.02	0.25
Q4	0.89	− 0.03	− 0.02	0.24
Q5	0.52	− 0.12	0.26	0.59
Q6	0.45	0.26	0.06	0.55
Q7	0.7	0.02	0.03	0.39
Q8	0.02	0.67	0.09	0.47
Q9	0.37	0.48	− 0.09	0.52
Q10	− 0.13	0.67	0.01	0.63
Q11	0.10	0.55	0.01	0.63

**Fig. 1:**
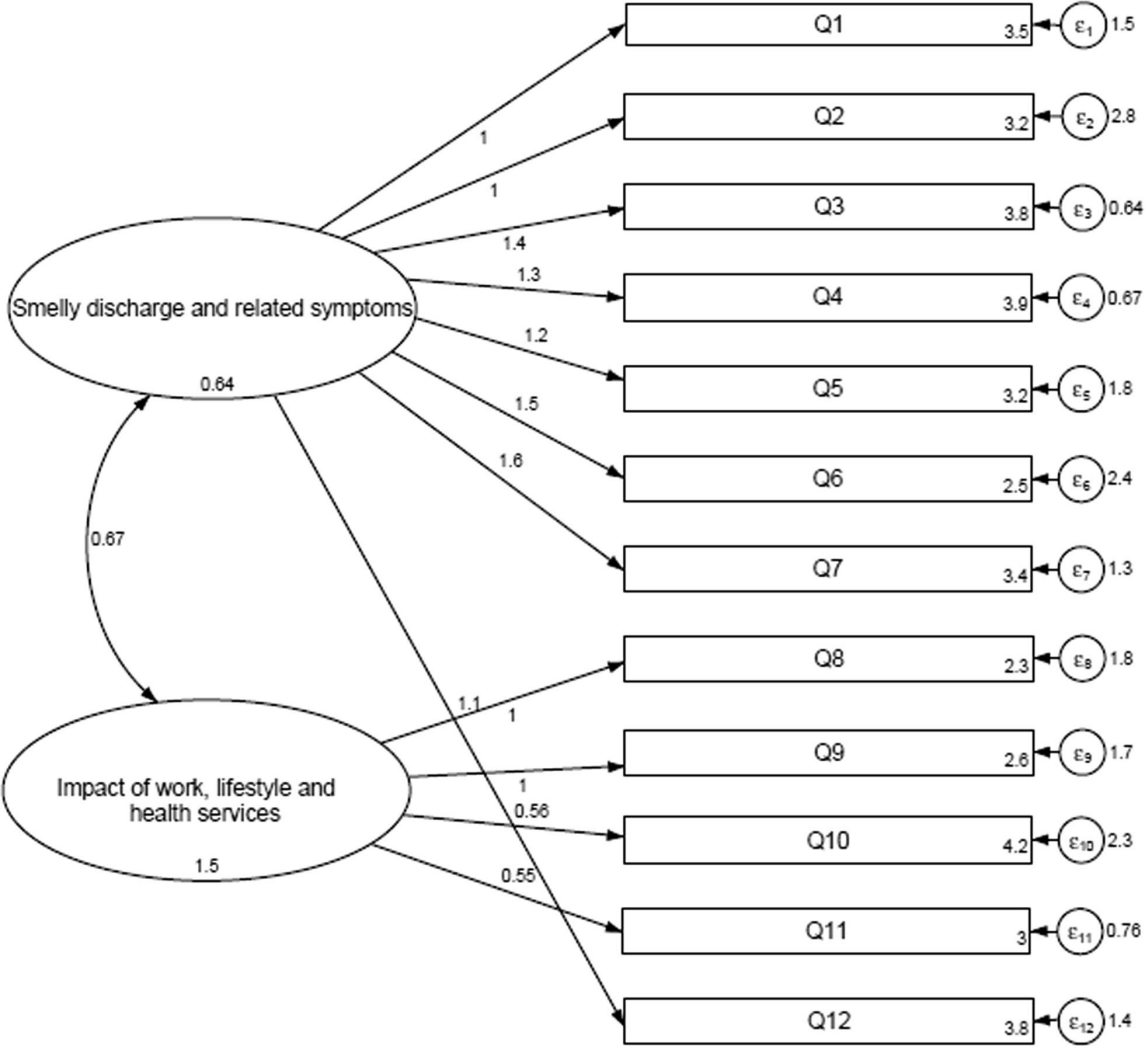
Exploratory factor analysis path (Model A). Variables are represented as rectangles, latent variables (factors) as ellipses and loading onto marker variables (COMQ-12 question items) as arrows. The error terms for the observable variables are shown as a circle plus arrow for each factor. Model A showed a two-factor structure and was obtained from an initial EFA

**Fig. 2:**
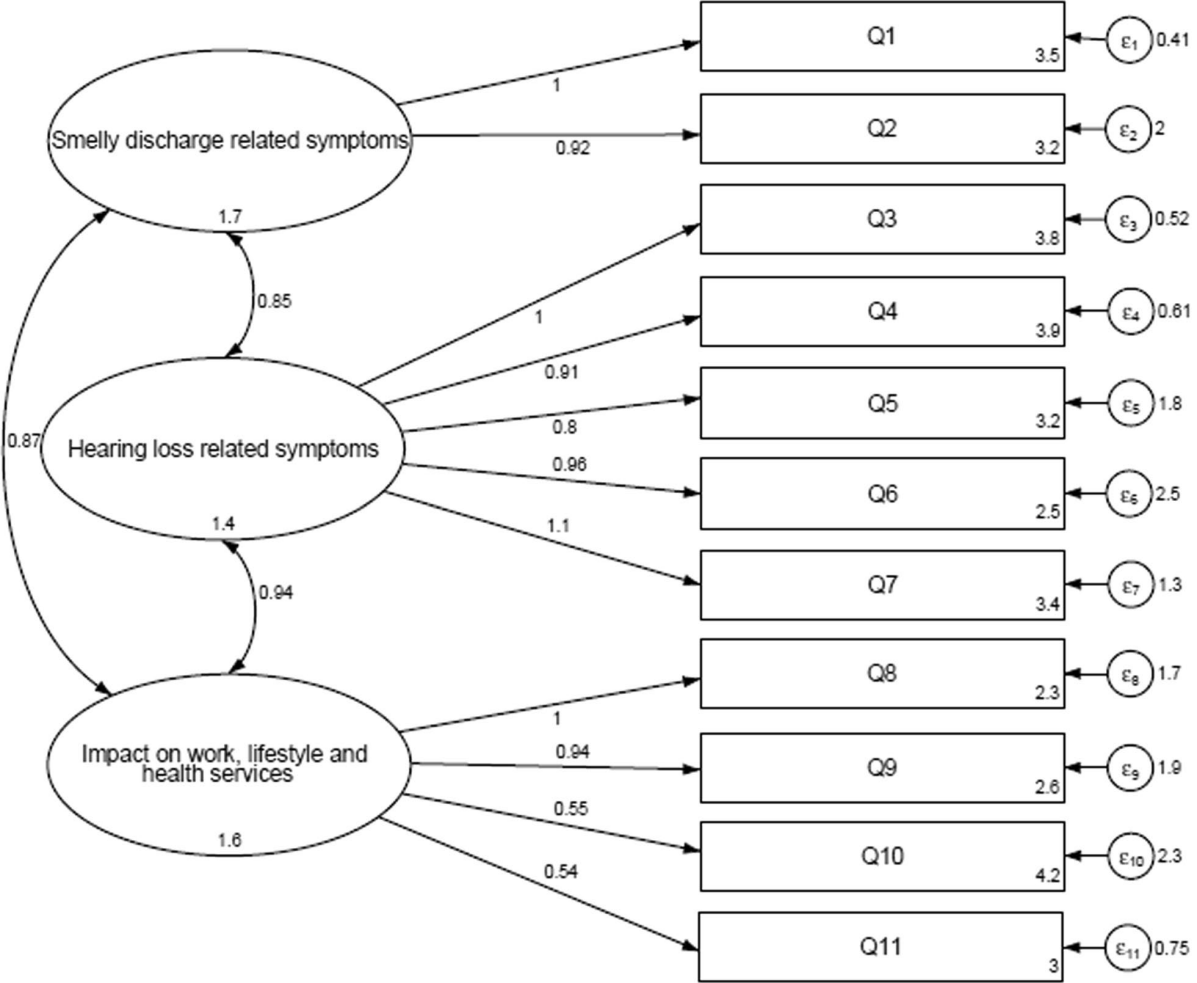
Confirmatory Factor Analysis path (Model B). Model B obtained from the CFA exhibited a three-factor structure. Variables are represented as rectangles, latent variables (factors) as ellipses and loading onto marker variables (COMQ-12 question items) as arrows. The error terms for the observable variables are shown as a circle plus arrow for each factor

CFA was performed and two different models were obtained: Model A (Fig. 1) showed a two-factor structure and was obtained from an initial EFA whereas Model B (Fig. 2) exhibited a three-factor structure. Figures 1 and 2 show the path diagram for both models and the corresponding associations between the observed and latent variables. The correlations between latent variables were calculated through a maximum likelihood estimation method using normalized latent variables.

Table 5 shows the goodness of fit values obtained for both models. Values obtained for Model B in the χ^2^/*df* ratio were closest to 1 and the RMSEA values were less than 0.06; those results are indicative of acceptable model fit. Likewise, Model B obtained the lowest SRMR values and a CFI value superior to 0.95; these outcomes supported the satisfactory fit of this model. As previously stated in the methodology section, the verification of the models obtained from the EFA and CFA were performed by splitting data into two halves (see Additional file 1). These results were consistent with prior EFA and CFA and supported the 3 domains model structure.

**Table 5:** The goodness of fit for both models

Indices	Model A (2 factors)	Model B (3 factors)
χ^2^	152.99	65.45
*p* value	0.001	0.001
*df*	66	41
χ^2^/*df*	2.31	1.56
RMSEA (IC 95%)	0.09 (0. 08; 0.12)	0.05 (0.02; 0.79)
TLI	0.85	0.95
CFI	0.88	0.97
SRMR	0.07	0.05
CD	0.95	0.99

### Concurrent criteria validity

Concurrent criteria validity was assessed through the COMQ-12 and VAS (Question 12 of the instrument) correlation. These results are shown in Table 6. The correlations ranged between 0.11 and 0.67. The best correlation coefficient was found between the domain "disease severity" and the VAS scale ($\widehat{\rho }$: 0.61; 95% CI 0.51; 0.69) (Table 6).

**Table 6:** COMQ-12 correlations with VAS score (12 items)

Correlation values with VAS score	Rho Spearman (*ρ*)	95% confidence interval	
Q1	0.43	0.31	0.54
Q2	0.41	0.29	0.52
Q3	0.51	0.39	0.60
Q4	0.52	0.41	0.62
Q5	0.42	0.30	0.53
Q6	0.44	0.32	0.54
Q7	0.44	0.32	0.54
Q8	0.26	0.12	0.38
Q9	0.53	0.42	0.62
Q10	0.11	-0.02	0.25
Q11	0.28	0.15	0.41
Symptom severity	0.61	0.51	0.69
Impact on lifestyle and work	0.48	0.36	0.58
Impact on health services	0.24	0.10	0.36
Total	0.68	0.59	0.74

### Validity

A random sample of 40 patients with COM was taken and compared with 31 healthy adults. Differences ranged between 4 points (general aspect domain) and 23 points (disease severity domain). A Wilcoxon Ranks test was performed to address the validity of the questionnaire. Statistically significant differences (*p* < 0.001*)* were found in all domains among patients with COM and healthy adults. The median difference and interquartile range (IQR) for each domain were as follows: for the “symptom severity” domain, a mean difference of 22 (IQR 17–25), for the “impact on lifestyle and work” domain, a mean difference of 3.5 (IQR 3–6.5), for the “impact on health services” domain, a mean difference of 7 (IQR 5.5–7), and for the “VAS” domain, a mean difference of 4 (IQR 3–5). These results support the questionnaire’s discriminating properties between COM patients and healthy adults.

Also, patients were classified into two groups regarding their clinical findings (medical history, otoscopic findings, and audiometry test results) to determine their disease activity: 76 patients had inactive COM, and 123 patients had active COM. The global score and domains were compared between these groups to assess the possible correlations (Table 7).

**Table 7:** Results of COMQ-12 domains according to disease activity

Disease activity	Domain	Median	IQR
Inactive COM (n:76)	Symptom severity	20.5	(14.5–26.5)
	Impact on lifestyle and work	4	(1–6.5)
	Impact on health services	8	(7–8.5)
	VAS	4	(3–5)
	Total	35	(26.5–45)
Active COM (n:123)	Symptom severity	27	(20.5–31.5)
	Impact on lifestyle and work	5	(3–8)
	Impact on health services	8	(7–8.5)
	VAS	4	(3–5)
	Total	44	(35–51.5)
Control group (n:31)	Symptom severity	3	(1–4)
	Impact on lifestyle and work	1	(0–1)
	Impact on health services	1	(1–2)
	VAS	0	(0–0)
	Total	5	(4–3)

Correlation coefficients between the activity of the disease and the domains of the questionnaire were calculated and ranged from 0.02 (health service impact) and 0.26 (symptom severity). Likewise, a Wilcoxon ranks test showed differences between patients with and without disease activity for the domains: symptom severity (*p* < 0.001), lifestyle and work impact (*p* < 0.05), and the total score (*p* < 0.05). No statistically significant differences were identified for the health service impact and general aspects domains.

## Discussion

The aim of this study was to validate the Spanish Language version of the COMQ-12 quality of life instrument (Additional file 2: Spanish version of the COMQ-12). The questionnaire showed strong psychometric properties such as high reliability and internal consistency, and coherent construct validity. Overall, most of the COMQ-12 items obtained a correlation level superior to 0.8, which means that each item significantly contributes to the global score of the questionnaire. Interestingly, the lowest correlation level was found with the dizziness item despite the prevalence and significance of this symptom in patients with COM that has been supported in the literature [1,41]. On the other hand, the highest correlation level was obtained by the items related to hearing loss, which is linked to limitations in communication that disturbs the quality of life in patients with COM [12].

Internal consistency was assessed using Cronbach’s alpha coefficient, which measures the homogeneity of items on a test [40]. Cronbach’s Alpha value for the Spanish version of the COMQ-12 was 0.86, similar to results published in previous validation studies [23]. Table 8 contains a summary of the internal consistency measures obtained with Cronbach’s Alpha for all COMQ-12 versions in different languages. Some psychometric experts propose that high Cronbach’s alpha values could lead to excessively narrow or even result in superficial measures, particularly if this is used as the only homogeneity criterion [42]. Moreover, a short tool like COMQ-12 designed to assess an outcome measure like QoL, could guarantees heterogeneity [9]. Nevertheless, the overall alpha values for the items of the questionnaire ranging from 0.82 to 0.86 could suggest that the questionnaire is sufficiently a coherent construct to be used in clinical settings.

**Table 8:** Internal consistency for different COMQ-12 versions

Questionnaire language	Participants (n)	Patients with COM (n)	Cronbach Alpha
Italian	48	48	0.80
English	50	50	0.89
Dutch	70	35	0.83
Portuguese	100	100	0.85
Kannada (India)	100	80	0.88
Turkish	100	50	0.81
Serbian	120	60	0.82
Russian	168	108	0.86
Korean	212	106	0.94
Spanish	231	200	0.86

Despite Cronbach’s Alpha being the most widely used coefficient in applied research for estimating reliability, its limitations are well known, such as the assumptions of uncorrelated errors (the error score of any pair of items is uncorrelated), tau-equivalence (equal factor loadings of all items in a factorial model), and normality which affects the reliability estimation [40]. Therefore, as an additional contribution, we calculated McDonald’s Omega coefficient, which is a robust measurement of internal consistency that corrects the underestimation bias of Cronbach’s Alpha when the tau-equivalence assumption is disrupted [43]. Different studies suggest that McDonald’s Omega coefficient is one of the best alternatives for estimating reliability [9,39,40]. McDonald’s Omega value obtained for the overall score was 0.89, which suggests a high degree of correlation between single items and the global score of the Spanish version of the COMQ-12.

Assessment of test re-test reliability values remained similar between the first and second visits (15 or 30 days after the first visit). The highest variation of the score was a mean difference not greater than one point for each domain. Although a statistically significant increase in the global score was found, ROC curves published in previous validations describe a cutoff point between 6 [24], 8 [21], and 9 [25] points to accurately discriminate between COM and control patients. Thus, a difference of one point in the global score in the second visit is not considered relevant in the clinical setting. Furthermore, Lin's Concordance correlation coefficient was 0.94, ranging between 0.93 and 0.96 for all domains, which demonstrates an outstanding test re-test correlation of the Spanish version of the COMQ-12. This value is higher than the value of 0.86 reported in the Dutch COMQ-12 [21], and lower than the Serbian version’s value of 0.98 [9]. Thus, compared to previous validations of different language versions of the COMQ-12, the Spanish version of COMQ-12 obtained similar empirical adequacy which is reflected in the test-re-test reliability and factor structure stability of the questionnaire.

On the other hand, factor analysis was performed to assess the internal structure of the Spanish COMQ-12 questionnaire. Two different models were identified according to eigenvalues and scree plot: Model A was obtained with the Exploratory Factor Analysis and two domains were extracted (Fig. 1); Model B was established by the Confirmatory Factor Analysis and three domains were defined (Fig. 2). Our findings suggested that Model A was similar to the model identified in the Korean validation of the COMQ-12 [17], and Model B was similar to the model proposed in the Serbian validation [9].

Hence, in order to compare the goodness of fit of both models, several statistical tests were conducted. According to scientific criteria, Model B obtained the best indices’ values, which support an acceptable and superior fit of this model. Parsimony, stability, and adequacy of Model B exhibited that this model was the most satisfactory one. Despite English, Serbian, and Spanish versions of the questionnaire have three domains, we found differences in item distribution and correlation strength indices. Thus, items in the Spanish version of the COMQ-12 are clustered in three domains: “smelly discharge related symptoms” (Q1 and Q2), “hearing loss related symptoms” (Q3–Q7) and “impact on work, lifestyle and health services” (Q8–Q11).

As previously reported, differences from the original English version of the COMQ-12 in item distribution and correlation strength indices could be explained by a “factor split” phenomenon which could be related to differences in health culture and health- care financing systems between countries [9]. As an illustration, the financial barrier of the Colombian healthcare system could cause a split between two roughly equal subsets of items loading on one existing factor, that is the division between the domain “impact on work, lifestyle” and “health services” (Q8–Q11) [9]. Likewise, comparing the reported alpha value of 0.82 obtained in the Serbian version of the COMQ-12 (which is similar to the Spanish version), with the values of 0.889 for the English version, we could argue that the English sample may be more homogeneous and so would be less likely to support a factor structure. These facts could affect the item distribution and correlation strength indices and could explain the differences between the versions of the COMQ-12 [9].

Regarding the concurrent validity of the test, correlation measurements between each item and the visual analogue scale or VAS (Q12) were done. Correlation values between the COMQ-12 global score and VAS were positive and closer to 1 (0.68), and the domain “symptom severity” obtained the higher correlation value ($\widehat{\rho }$: 0.61; 95% CI 0.51–0.69). These findings were similarly described in previously published validations [9,24,26]. Thus, as the symptom severity score increases, a greater VAS score should be obtained.

Finally, Wilcoxon ranks test addressed the validity of the questionnaire. Statistically significant differences were found in the COMQ-12 global score between COM and control patients (*p* < 0.0001). This finding suggests that the Spanish version of the COMQ-12 can accurately discriminate between COM and healthy adults, similar to Dutch, Serbian, Russian, and Korean versions [9,21,23,24]. Likewise, a correlation analysis was also performed classifying COM patients according to their disease activity based on the otoscopic findings. Patients with active disease (active squamous epithelium or cholesteatoma and perforated eardrum with discharge) had a greater overall score with statistically significant differences in “symptom severity” and “lifestyle and work impact” domains. These results are similar to previous COMQ-12 validations [1,9,23,24], and therefore, confirm the relationship between questionnaire score and disease activity.

In conclusion, considering the high impact of COM on patients’ QoL, this questionnaire can be used as a routine clinical tool to assess HRQoL outcomes in Hispanic patients. Incorporating this tool in clinical settings would definitively help ear, nose, and throat specialists to understand the impairment in quality of life of their patients and would guide them to provide appropriate management with a patient-centric approach.

### Strengths and limitations

One of the strengths of this study is the sample size, which has the biggest number of patients with COM compared to previous COMQ-12 validation studies. Also, the otoscopic examination of every patient enrolled was confirmed by two neuro-otologists. Therefore, reliable and homogeneous statistic results were obtained. Likewise, we have addressed our statistical models via exploratory factor analysis, and rigorous statistical strategies for extraction and interpretation were performed. Since it is not clear how the questionnaire should be applied (i.e. independent or assisted) [9], a trained medical doctor and two otologists were available to provided minimum assistance only if the subjects explicitly asked for it through the questionnaire. This aspect should be considered as an important limitation. Besides, previous psychometric guidelines for the validation of QoL instruments suggest that several pilot tests should be used to validate a questionnaire. We performed one pilot test to validate our instrument, thus this is another relevant limitation of this study. Finally, we did not measure the responsiveness of the questionnaire due to surgical intervention. However, this statistical property has not been reported in any previous COMQ-12 validation yet, and we expect to assess it in the second phase of this study.

## Conclusion

The Spanish version of the COMQ-12 showed high reliability and high internal consistency. This questionnaire is an objective clinical tool that assesses HRQoL in patients with COM diagnosis. Factor analysis of the main components of the Spanish version of COMQ-12 indicated a structure with three domains: smelly discharge related symptoms; hearing loss related symptoms; and impact on work, lifestyle, and health services.

## Supplementary information

**Additional file 1**. Factor analysis performed to verify EFA and a CFA using a randomly split sample in two halves.

**Additional file 2**. Spanish version of the Chronic Otitis Media Questionnaire (COMQ-12).

## Data Availability

Part of the data generated or analyzed during this study is included in this published article (and its additional files). Full datasets generated during and/or analyzed during the current study are available from the corresponding author on reasonable request.

## Abbreviations

CD: Coefficient of determination
CFA: Confirmatory Factor Analysis
CFI: Bentler’s Comparative Fit index
COM: Chronic Otitis Media
COMQ-12: Chronic Otitis Media Questionnaire 12
DALYs: Disability-Adjusted Life Years
EORTC: European Organization for Research and Treatment of Cancer
EFA: Exploratory Factor Analysis
HRQoL: Health-related quality of life
KMO: Kaiser–Meyer–Olkin
RMSEA: Root mean square error approximation
SD: Standard deviation
SEM: Structural equation modeling
SRMR: Standardized root mean square residual
TLI: Tucker–Lewis index
VAS: Visual Analogue Scale
