# Supplementary material for: Validation of a Spanish version of the health-related quality of life (HRQoL) measure for Chronic Otitis Media (COMQ-12)
Source: Health Qual Life Outcomes. 2020 Nov 10;18:362. doi: 10.1186/s12955-020-01616-5 (PMC7654037; doi:10.1186/s12955-020-01616-5)
Supplement: Supplementary file 1 — Additional file 1. Factor analysis performed to verify EFA and a CFA using a randomly split sample in two halves. [file 12955_2020_1616_MOESM1_ESM.pdf]

# ESCALA COMQ-12

***Cuestionario de Otitis Media Crónica (COMQ-12):***

Estas preguntas tienen por objeto averiguar qué tanto lo afecta su problema del oído. Ningún aparato puede hacerlo: solo usted puede explicarnos cómo se siente. Esperamos que los resultados de este cuestionario nos ayuden a comprender cuál de los síntomas en su oído es el más importante para usted. Al saber esto podemos mejorar las formas de atención a los pacientes con problemas del oído.

Por favor responda las siguientes preguntas considerando cuidadosamente cada pregunta y marcando el número correspondiente. Cada número se refiere a una descripción específica. No hay respuestas correctas ni incorrectas. Simplemente trate de pensar cuidadosamente cada pregunta antes de marcar el número que considere apropiado. Considere cada problema y responda si ha sentido alguno de estos síntomas durante ***LOS ÚLTIMOS 6 MESES.***

**EJEMPLO:**

Para la siguiente pregunta por favor indique con qué frecuencia usted realiza esta actividad aplicando la escala indicada y marcando el número correspondiente con una X:

| **EN LOS ULTIMOS 6 MESES:** | **nunca** | **al menos una vez cada 3 meses** | **al menos una vez al mes** | **al menos una vez a la semana** | **casi todos los días de la semana** | **todo el tiempo** |
|---|---|---|---|---|---|---|
| | **0** | **1** | **2** | **3** | **4** | **5** |
| **¿Con que frecuencia se come una tostada al desayuno?** | | | X | | | |

Una persona que responde así dice que generalmente se come una tostada al desayuno, al menos una vez al mes. Si usted tiene algún problema para responder a las preguntas por favor pídale ayuda a algún miembro del personal de la clínica.

**Gracias.**

## SEVERIDAD DE LOS SÍNTOMAS

Para las siguientes preguntas por favor indique que tanto lo afectaron a usted los siguientes síntomas. Use la escala descrita a continuación y marque el número que corresponda con una equis **(X)**.

| **EN LOS ULTIMOS 6 MESES Que tanto le molestó o afectó:** | **no me afectó o no me molestó para nada** | **fue una molestia menor** | **fue una molestia moderada** | **fue una molestia grave, pero la pude manejar** | **fue una molesta grave y me costó trabajo manejarla** | **la peor cosa que me ha afectado en mi vida** |
|---|---|---|---|---|---|---|
| | **0** | **1** | **2** | **3** | **4** | **5** |
| **1.** Las secreciones o el drenaje del oído | | | | | | |
| **2.** El mal olor del oído | | | | | | |
| **3.** Los problemas para oír en la casa que requieren, por ejemplo, que suba el volumen de la TV o del radio | | | | | | |
| **4.** Los problemas para oír cuando hablo con varias personas o en grupos o cuando el medio ambiente es ruidoso | | | | | | |
| **5.** Molestias dentro y alrededor del oído | | | | | | |
| **6.** Los mareos o sensación de "pérdida del equilibrio" | | | | | | |
| **7.** Tinnitus o ruidos en el oído | | | | | | |

## ASPECTOS GENERALES

Para las siguientes preguntas por favor indique que ***tan mal se ha sentido en su estado de ánimo.*** En una escala de 0 a 5 donde 0 significa que no le molesta y 5 significa que se ha sentido muy mal.

**EN LOS ULTIMOS 6 MESES:**
**Que tan mal se ha sentido en su estado de ánimo**

Hasta qué punto sus problemas del oído lo "han hecho sentir mal". Marque con una equis **(X)** la opción que corresponda

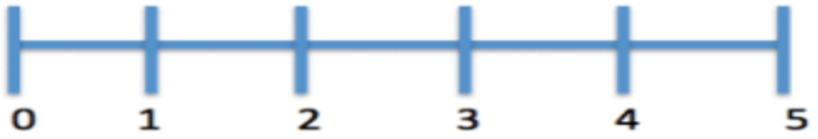


No me Molesta

Me he sentido Muy mal

## IMPACTO EN EL ESTILO DE VIDA Y EL TRABAJO

***Para las siguientes preguntas*** por favor indique ***que tan frecuente o con qué frecuencia*** lo afectaron a usted los siguientes problemas.

Use la escala descrita abajo y marque el número que corresponda con una equis **(X)**

| **EN LOS ULTIMOS 6 MESES Con qué frecuencia, por su problema de oído, usted NO pudo:** | ***NINGUNA VEZ* en los últimos *6 MESES*** | ***POR LO MENOS UNA VEZ* en los últimos *6 MESES*** | ***POR LO MENOS UNA VEZ* en los últimos *3 MESES*** | ***POR LO MENOS UNA VEZ* en el último *MES*** | ***POR LO MENOS UNA VEZ por SEMANA*** | **la mayor parte *DE LOS DÍAS DE LA SEMANA*** |
|---|---|---|---|---|---|---|
| | **0** | **1** | **2** | **3** | **4** | **5** |
| **1.** ¿Realizar sus actividades diarias normales en casa o en el trabajo? | | | | | | |
| **2.** Debido a su problema en el oído, ¿con qué frecuencia o que tanto a usted le da miedo bañarse, ducharse o realizar actividades en el agua por miedo a una infección en el oído? | | | | | | |

## IMPACTO EN EL SERVICIO DE SALUD

***Para las siguientes preguntas*** por favor indique ***que tan frecuente o con qué frecuencia*** lo afectaron a usted los siguientes problemas.

Use la escala descrita abajo y marque el número que corresponda con una equis **(X)**

| **EN LOS ULTIMOS 6 MESES (Impacto en el Servicio de Salud)** | ***NINGUNA VEZ* en los últimos *6 MESES*** | ***POR LO MENOS UNA VEZ* en los últimos *6 MESES*** | ***POR LO MENOS UNA VEZ* en los últimos *3 MESES*** | ***POR LO MENOS UNA VEZ* en el último *MES*** | ***POR LO MENOS UNA VEZ por SEMANA*** | **la mayor parte *DE LOS DÍAS DE LA SEMANA*** |
|---|---|---|---|---|---|---|
| | **0** | **1** | **2** | **3** | **4** | **5** |
| **1.** ¿Con qué frecuencia o que tanto visitó a su médico a causa de los problemas en su oído?: | | | | | | |
| **2.** ¿Con qué frecuencia o que tanto tiene que tomar medicamentos (incluyendo gotas para el oído) para sus problemas del oído? | | | | | | |
