# Supplementary material for: Validation of a Spanish version of the health-related quality of life (HRQoL) measure for Chronic Otitis Media (COMQ-12)
Source: Health Qual Life Outcomes. 2020 Nov 10;18:362. doi: 10.1186/s12955-020-01616-5 (PMC7654037; doi:10.1186/s12955-020-01616-5)
Supplement: Supplementary file 2 — Additional file 2. Spanish version of the Chronic Otitis Media Questionnaire (COMQ-12). [file 12955_2020_1616_MOESM2_ESM.pdf]

```
------------------------------------------------------------------------------------------------------------------------
      name:  <unnamed>
       log:  C:\Users\smmor\Desktop\Analisis factorial adicional COMQ-12 03 jul 2020.log
  log type:  text
 opened on:   3 Jul 2020, 18:12:32

. 
. ******FACTOR ANALISIS SAMPLE SIZE COMPLETE
. 
. *bartlet test
. 
. factortest pre1lassecrecionesoeldrena - pre11conquéfrecuenciaoque

Determinant of the correlation matrix
Det                 =     0.021


Bartlett test of sphericity

Chi-square          =           749.170
Degrees of freedom =                55
p-value             =             0.000
H0: variables are not intercorrelated


Kaiser-Meyer-Olkin Measure of Sampling Adequacy
KMO               =     0.849

. 
. *policóric Matrix
. 
. polychoric pre1lassecrecionesoeldrena - pre11conquéfrecuenciaoque

Polychoric correlation matrix

                              pre1lassecrecionesoeldrena       pre2elmalolordeloído   pre3losproblemasparaoíren
 pre1lassecrecionesoeldrena                            1
      pre2elmalolordeloído                     .66275044                           1
 pre3losproblemasparaoíren                     .50400075                   .33879378                           1
pre4losproblemasparaoírcua                     .43312827                    .3735871                   .79870284
pre5molestiasdentroyalreded                    .43810102                   .35581485                   .52326945
 pre6losmareososensaciónde                     .37977622                   .33432019                   .52977376
 pre7tinnitusoruidoseneloí                      .4054954                    .3414366                    .6800446
  pre8conquéfrecuenciaoque                     .43819377                   .27042993                    .3792038
 pre9realizarsusactividades                    .32722622                   .28191422                   .47825503
  pre10debidoasuproblemaene                      .255308                   .13884614                   .21281281
 pre11conquéfrecuenciaoque                     .30961306                   .24355824                   .35700507

                             pre4losproblemasparaoírcua  pre5molestiasdentroyalreded   pre6losmareososensaciónde
pre4losproblemasparaoírcua                            1
pre5molestiasdentroyalreded                    .51645089                           1
 pre6losmareososensaciónde                     .49653723                   .41998747                           1
 pre7tinnitusoruidoseneloí                     .66035203                   .51180435                   .55420548
  pre8conquéfrecuenciaoque                     .35319519                   .23301642                   .37952411
 pre9realizarsusactividades                    .55065807                   .23804182                   .45616676
  pre10debidoasuproblemaene                    .11660919                   .14539798                   .36627558
 pre11conquéfrecuenciaoque                     .34804371                   .20232351                   .27865644

                              pre7tinnitusoruidoseneloí    pre8conquéfrecuenciaoque   pre9realizarsusactividades
 pre7tinnitusoruidoseneloí                            1
  pre8conquéfrecuenciaoque                     .34800812                           1
 pre9realizarsusactividades                    .43167115                    .4750009                           1
  pre10debidoasuproblemaene                     .1840132                   .47389529                   .31376183
 pre11conquéfrecuenciaoque                      .3169296                   .48236896                   .44417918

                               pre10debidoasuproblemaene   pre11conquéfrecuenciaoque
  pre10debidoasuproblemaene                            1
 pre11conquéfrecuenciaoque                      .3520045                           1

. 
. matrix poly=r(R)
. 
. *EFA without q12
. 
. factormat poly, n(200) factor(3)
(obs=200)

Factor analysis/correlation                        Number of obs    =        200
    Method: principal factors                      Retained factors =          3
    Rotation: (unrotated)                          Number of params =         30

    --------------------------------------------------------------------------
         Factor  |   Eigenvalue   Difference        Proportion   Cumulative
    -------------+------------------------------------------------------------
        Factor1  |      4.53265      3.72416            0.8357       0.8357
        Factor2  |      0.80849      0.22228            0.1491       0.9848
        Factor3  |      0.58620      0.39120            0.1081       1.0928
        Factor4  |      0.19500      0.14025            0.0360       1.1288
        Factor5  |      0.05476      0.08367            0.0101       1.1389
        Factor6  |     -0.02892      0.04257           -0.0053       1.1335
```

```
       Factor7  |     -0.07149      0.03531           -0.0132       1.1204
       Factor8  |     -0.10680      0.04203           -0.0197       1.1007
       Factor9  |     -0.14883      0.03955           -0.0274       1.0732
      Factor10  |     -0.18837      0.02047           -0.0347       1.0385
      Factor11  |     -0.20884            .           -0.0385       1.0000
    --------------------------------------------------------------------------
    LR test: independent vs. saturated:  chi2(55) = 1012.53 Prob>chi2 = 0.0000

Factor loadings (pattern matrix) and unique variances

    -----------------------------------------------------------
        Variable |  Factor1   Factor2   Factor3 |   Uniqueness 
    -------------+------------------------------+--------------
    pre1lassec~a |   0.6693    0.0333    0.4610 |      0.3384  
    pre2elmalo~o |   0.5458   -0.0347    0.4841 |      0.4665  
    pre3lospro~n |   0.8147   -0.2511   -0.1568 |      0.2486  
    pre4lospro~a |   0.7975   -0.3001   -0.1858 |      0.2394  
    pre5molest~d |   0.5859   -0.2444    0.0914 |      0.5886  
    pre6losmar~e |   0.6650    0.0286   -0.0834 |      0.5500  
    pre7tinnit~í |   0.7347   -0.2167   -0.1321 |      0.3959  
    pre8conqué~e |   0.5890    0.4258   -0.0233 |      0.4712  
    pre9realiz~s |   0.6350    0.1981   -0.1949 |      0.5196  
    pre10debid~e |   0.3884    0.4698   -0.0481 |      0.6261  
    pre11conqu~e |   0.5105    0.3230   -0.0817 |      0.6284  
    -----------------------------------------------------------

.
. rotate, factor(3) blank(0.25) varimax

Factor analysis/correlation                      Number of obs    =        200
    Method: principal factors                    Retained factors =          3
    Rotation: orthogonal varimax (Kaiser off)    Number of params =         30

    --------------------------------------------------------------------------
         Factor  |     Variance   Difference        Proportion   Cumulative
    -------------+------------------------------------------------------------
        Factor1  |      3.01875      1.47811            0.5566       0.5566
        Factor2  |      1.54063      0.17267            0.2840       0.8406
        Factor3  |      1.36796            .            0.2522       1.0928
    --------------------------------------------------------------------------
    LR test: independent vs. saturated:  chi2(55) = 1012.53 Prob>chi2 = 0.0000

Rotated factor loadings (pattern matrix) and unique variances

    -----------------------------------------------------------
        Variable |  Factor1   Factor2   Factor3 |   Uniqueness 
    -------------+------------------------------+--------------
    pre1lassec~a |   0.3265              0.7109 |      0.3384  
    pre2elmalo~o |   0.2559              0.6762 |      0.4665  
    pre3lospro~n |   0.8181                     |      0.2486  
    pre4lospro~a |   0.8404                     |      0.2394  
    pre5molest~d |   0.5424              0.3412 |      0.5886  
    pre6losmar~e |   0.5336    0.3409           |      0.5500  
    pre7tinnit~í |   0.7293                     |      0.3959  
    pre8conqué~e |   0.2525    0.6369           |      0.4712  
    pre9realiz~s |   0.4680    0.4995           |      0.5196  
    pre10debid~e |             0.5910           |      0.6261  
    pre11conqu~e |   0.2654    0.5262           |      0.6284  
    -----------------------------------------------------------
    (blanks represent abs(loading)<.25)

Factor rotation matrix

    -----------------------------------------
                 | Factor1  Factor2  Factor3 
    -------------+---------------------------
         Factor1 |  0.7760   0.4469   0.4451 
         Factor2 | -0.5016   0.8651   0.0059 
         Factor3 | -0.3824  -0.2278   0.8955 
    -----------------------------------------

.
. rotate, promax horst blanks(.3)

Factor analysis/correlation                      Number of obs    =        200
    Method: principal factors                    Retained factors =          3
    Rotation: oblique promax (Kaiser on)         Number of params =         30

    --------------------------------------------------------------------------
         Factor  |     Variance   Proportion    Rotated factors are correlated
    -------------+------------------------------------------------------------
        Factor1  |      4.01959       0.7411
        Factor2  |      2.91809       0.5380
        Factor3  |      2.65472       0.4895
    --------------------------------------------------------------------------
    LR test: independent vs. saturated:  chi2(55) = 1012.53 Prob>chi2 = 0.0000

Rotated factor loadings (pattern matrix) and unique variances

    -----------------------------------------------------------
        Variable |  Factor1   Factor2   Factor3 |   Uniqueness 
```

```
    -------------+------------------------------+--------------
    pre1lassec~a |                      0.7102 |      0.3384
    pre2elmalo~o |                      0.7121 |      0.4665
    pre3lospro~n |   0.8467                     |      0.2486
    pre4lospro~a |   0.8984                     |      0.2394
    pre5molest~d |   0.5243                     |      0.5886
    pre6losmar~e |   0.4567                     |      0.5500
    pre7tinnit~í |   0.7486                     |      0.3959
    pre8conqué~e |            0.6704            |      0.4712
    pre9realiz~s |   0.3665    0.4803           |      0.5196
    pre10debid~e |            0.6650            |      0.6261
    pre11conqu~e |            0.5478            |      0.6284
    -----------------------------------------------------------
    (blanks represent abs(loading)<.3)

Factor rotation matrix

    -----------------------------------------
                 | Factor1  Factor2  Factor3
    -------------+---------------------------
         Factor1 |  0.9298   0.7532   0.7240
         Factor2 | -0.3104   0.6465  -0.0139
         Factor3 | -0.1977  -0.1211   0.6897
    -----------------------------------------
.
. matrix factor=e(r_L)
.
. **Annexes
.
. *Comunality
.
. estat smc

Squared multiple correlations of variables with all other variables

    -----------------------
        Variable |     smc
    -------------+---------
    pre1lassec~a |  0.5764
    pre2elmalo~o |  0.4732
    pre3lospro~n |  0.7156
    pre4lospro~a |  0.7167
    pre5molest~d |  0.3871
    pre6losmar~e |  0.4496
    pre7tinnit~í |  0.5591
    pre8conqué~e |  0.4445
    pre9realiz~s |  0.4539
    pre10debid~e |  0.3233
    pre11conqu~e |  0.3245
    -----------------------
.
. *Goodness of fit

.
. estat factors, factors(3)

Factor analysis with different numbers of factors (maximum likelihood)

    ----------------------------------------------------------
    #factors |     loglik   df_m   df_r        AIC        BIC
    ---------+------------------------------------------------
           1 |   -125.829     11     44   273.6579   309.9394
           2 |  -68.22127     21     34   178.4425   247.7072
           3 |  -25.50172     30     25   111.0034    209.953
    ----------------------------------------------------------
    the model with 3 factors is a Heywood case
.
. *Correltaion Matrrix domains
.
. estat common

Correlation matrix of the promax(3) rotated common factors

    --------------------------------------------
         Factors |  Factor1   Factor2   Factor3
    -------------+------------------------------
         Factor1 |        1
         Factor2 |    .5236         1
         Factor3 |    .5411     .4528         1
    --------------------------------------------

. **CFA

. sem (Secrecionymalolor -> pre1lassecrecionesoeldrena, ) (Secrecionymalolor -> pre2elmalolordeloído, ) (Problemasauditivos ->
> pre3losproblemasparaoíren, ) (Problemasauditivos -> pre4losproblemasparaoírcua, ) (Problemasauditivos -> pre5molestiasdentroy
> alreded, ) (Problemasauditivos -> pre6losmareososensaciónde, ) (Problemasauditivos -> pre7tinnitusoruidoseneloí, ) (Impactosa
> ludestilovida -> pre8conquéfrecuenciaoque, ) (Impactosaludestilovida -> pre9realizarsusactividades, ) (Impactosaludestilovida
>  -> pre10debidoasuproblemaene, ) (Impactosaludestilovida -> pre11conquéfrecuenciaoque, ), covstruct(_lexogenous, diagonal) la
> tent(Secrecionymalolor Problemasauditivos Impactosaludestilovida ) cov( Secrecionymalolor*Problemasauditivos Problemasauditiv
> os*Impactosaludestilovida Impactosaludestilovida*Secrecionymalolor) nocapslatent
```

```
Endogenous variables

Measurement:  pre1lassecrecionesoeldrena pre2elmalolordeloído pre3losproblemasparaoíren pre4losproblemasparaoírcua
              pre5molestiasdentroyalreded pre6losmareososensaciónde pre7tinnitusoruidoseneloí pre8conquéfrecuenciaoque
              pre9realizarsusactividades pre10debidoasuproblemaene pre11conquéfrecuenciaoque

Exogenous variables

Latent:       Secrecionymalolor Problemasauditivos Impactosaludestilovida

Fitting target model:

Iteration 0:   log likelihood = -3791.8767
Iteration 1:   log likelihood =  -3789.573
Iteration 2:   log likelihood = -3789.5228
Iteration 3:   log likelihood = -3789.5227

Structural equation model                       Number of obs     =        200
Estimation method  = ml
Log likelihood     = -3789.5227

 ( 1)  [pre1lassecrecionesoeldrena]Secrecionymalolor = 1
 ( 2)  [pre3losproblemasparaoíren]Problemasauditivos = 1
 ( 3)  [pre8conquéfrecuenciaoque]Impactosaludestilovida = 1
------------------------------------------------------------------------------------------------------------------------
                                             |                 OIM
                                             |      Coef.   Std. Err.      z    P>|z|     [95% Conf. Interval]
---------------------------------------------+--------------------------------------------------------------------------
Measurement                                  |
  pre1lassecrecionesoeldrena                 |
                           Secrecionymalolor |          1  (constrained)
                                       _cons |      3.505   .1034401    33.88   0.000     3.302261    3.707739
  -------------------------------------------+--------------------------------------------------------------------------
  pre2elmalolordeloído                       |
                           Secrecionymalolor |   .9226591   .1484445     6.22   0.000     .6317133    1.213605
                                       _cons |       3.23   .1316641    24.53   0.000     2.971943    3.488057
  -------------------------------------------+--------------------------------------------------------------------------
  pre3losproblemasparaoíren                  |
                          Problemasauditivos |          1  (constrained)
                                       _cons |       3.82   .0971493    39.32   0.000     3.629591    4.010409
  -------------------------------------------+--------------------------------------------------------------------------
  pre4losproblemasparaoírcua                 |
                          Problemasauditivos |   .9104383   .0690864    13.18   0.000     .7750314    1.045845
                                       _cons |       3.91   .0933247    41.90   0.000     3.727087    4.092913
  -------------------------------------------+--------------------------------------------------------------------------
  pre5molestiasdentroyalreded                |
                          Problemasauditivos |   .7976832    .098145     8.13   0.000     .6053226    .9900439
                                       _cons |        3.2   .1159741    27.59   0.000     2.972695    3.427305
  -------------------------------------------+--------------------------------------------------------------------------
  pre6losmareososensaciónde                  |
                          Problemasauditivos |   .9636699   .1183889     8.14   0.000     .7316319    1.195708
                                       _cons |      2.525   .1381914    18.27   0.000      2.25415     2.79585
  -------------------------------------------+--------------------------------------------------------------------------
  pre7tinnitusoruidoseneloí                  |
                          Problemasauditivos |   1.073937   .0969934    11.07   0.000     .8838334    1.264041
                                       _cons |      3.415   .1208878    28.25   0.000     3.178064    3.651936
  -------------------------------------------+--------------------------------------------------------------------------
  pre8conquéfrecuenciaoque                   |
                      Impactosaludestilovida |          1  (constrained)
                                       _cons |       2.27   .1287847    17.63   0.000     2.017587    2.522413
  -------------------------------------------+--------------------------------------------------------------------------
  pre9realizarsusactividades                 |
                      Impactosaludestilovida |   .9373332   .1411192     6.64   0.000     .6607446    1.213922
                                       _cons |      2.635   .1279018    20.60   0.000     2.384317    2.885683
  -------------------------------------------+--------------------------------------------------------------------------
  pre10debidoasuproblemaene                  |
                      Impactosaludestilovida |   .5520066   .1092097     5.05   0.000     .3379596    .7660536
                                       _cons |       4.16   .1179915    35.26   0.000     3.928741    4.391259
  -------------------------------------------+--------------------------------------------------------------------------
  pre11conquéfrecuenciaoque                  |
                      Impactosaludestilovida |    .539203   .0786025     6.86   0.000     .3851449    .6932611
                                       _cons |       3.03   .0780737    38.81   0.000     2.876978    3.183022
---------------------------------------------+--------------------------------------------------------------------------
             var(e.pre1lassecrecionesoeldrena)|   .4071099   .2456579                          .1247615    1.328443
                   var(e.pre2elmalolordeloído)|   1.991902   .2867224                          1.502252    2.641152
              var(e.pre3losproblemasparaoíren)|   .5201279   .0841954                          .3787236    .7143284
             var(e.pre4losproblemasparaoírcua)|   .6084056   .0829949                          .4656693    .7948932
            var(e.pre5molestiasdentroyalreded)|    1.81988   .1956379                          1.474138    2.246711
              var(e.pre6losmareososensaciónde)|   2.549459   .2767624                          2.060838    3.153931
              var(e.pre7tinnitusoruidoseneloí)|   1.345614   .1636353                          1.060252    1.707781
               var(e.pre8conquéfrecuenciaoque)|   1.715977   .2560137                          1.280905    2.298824
             var(e.pre9realizarsusactividades)|   1.865038   .2554206                          1.425982    2.439279
              var(e.pre10debidoasuproblemaene)|    2.29652   .2473396                          1.859491    2.836261
              var(e.pre11conquéfrecuenciaoque)|   .7535895   .0943918                          .5895444    .9632813
                        var(Secrecionymalolor)|   1.732862   .3206668                          1.205728    2.490455
                       var(Problemasauditivos)|   1.367471   .1931539                           1.03678    1.803638
                   var(Impactosaludestilovida)|   1.601123   .3415889                          1.053966    2.432332
---------------------------------------------+--------------------------------------------------------------------------
     cov(Secrecionymalolor,Problemasauditivos)|   .8469176    .146986     5.76   0.000     .5588303    1.135005
```

```
 cov(Secrecionymalolor,Impactosaludestilovida)|   .8709498   .1832529     4.75   0.000     .5117807    1.230119
cov(Problemasauditivos,Impactosaludestilovida)|   .9404762    .164866     5.70   0.000     .6173447    1.263608
-----------------------------------------------------------------------------------------------------------------
LR test of model vs. saturated: chi2(41)  =     65.45, Prob > chi2 = 0.0090

.
. estimate store modelo3fact

.
. estat gof, stats(all)

----------------------------------------------------------------------------
Fit statistic        |      Value   Description
---------------------+------------------------------------------------------
Likelihood ratio     |
         chi2_ms(41) |     65.445   model vs. saturated
            p > chi2 |      0.009
         chi2_bs(55) |    770.355   baseline vs. saturated
            p > chi2 |      0.000
---------------------+------------------------------------------------------
Population error     |
               RMSEA |      0.055   Root mean squared error of approximation
 90% CI, lower bound |      0.028
         upper bound |      0.079
              pclose |      0.354   Probability RMSEA <= 0.05
---------------------+------------------------------------------------------
Information criteria |
                 AIC |   7651.045   Akaike's information criterion
                 BIC |   7769.785   Bayesian information criterion
---------------------+------------------------------------------------------
Baseline comparison  |
                 CFI |      0.966   Comparative fit index
                 TLI |      0.954   Tucker-Lewis index
---------------------+------------------------------------------------------
Size of residuals    |
                SRMR |      0.050   Standardized root mean squared residual
                  CD |      0.989   Coefficient of determination
----------------------------------------------------------------------------

. factormat poly, n(200) factor(3)
(obs=200)

Factor analysis/correlation                      Number of obs    =        200
    Method: principal factors                    Retained factors =          3
    Rotation: (unrotated)                        Number of params =         30

    --------------------------------------------------------------------------
         Factor  |   Eigenvalue   Difference        Proportion   Cumulative
    -------------+------------------------------------------------------------
        Factor1  |      4.53265      3.72416            0.8357       0.8357
        Factor2  |      0.80849      0.22228            0.1491       0.9848
        Factor3  |      0.58620      0.39120            0.1081       1.0928
        Factor4  |      0.19500      0.14025            0.0360       1.1288
        Factor5  |      0.05476      0.08367            0.0101       1.1389
        Factor6  |     -0.02892      0.04257           -0.0053       1.1335
        Factor7  |     -0.07149      0.03531           -0.0132       1.1204
        Factor8  |     -0.10680      0.04203           -0.0197       1.1007
        Factor9  |     -0.14883      0.03955           -0.0274       1.0732
       Factor10  |     -0.18837      0.02047           -0.0347       1.0385
       Factor11  |     -0.20884            .           -0.0385       1.0000
    --------------------------------------------------------------------------
    LR test: independent vs. saturated:  chi2(55) = 1012.53 Prob>chi2 = 0.0000

Factor loadings (pattern matrix) and unique variances

    -----------------------------------------------------------
        Variable |  Factor1   Factor2   Factor3 |   Uniqueness
    -------------+------------------------------+--------------
    pre1lassec~a |   0.6693    0.0333    0.4610 |      0.3384
    pre2elmalo~o |   0.5458   -0.0347    0.4841 |      0.4665
    pre3lospro~n |   0.8147   -0.2511   -0.1568 |      0.2486
    pre4lospro~a |   0.7975   -0.3001   -0.1858 |      0.2394
    pre5molest~d |   0.5859   -0.2444    0.0914 |      0.5886
    pre6losmar~e |   0.6650    0.0286   -0.0834 |      0.5500
    pre7tinnit~í |   0.7347   -0.2167   -0.1321 |      0.3959
    pre8conqué~e |   0.5890    0.4258   -0.0233 |      0.4712
    pre9realiz~s |   0.6350    0.1981   -0.1949 |      0.5196
    pre10debid~e |   0.3884    0.4698   -0.0481 |      0.6261
    pre11conqu~e |   0.5105    0.3230   -0.0817 |      0.6284
    -----------------------------------------------------------

.
. rotate, factor(3) blank(0.25) varimax

Factor analysis/correlation                      Number of obs    =        200
    Method: principal factors                    Retained factors =          3
    Rotation: orthogonal varimax (Kaiser off)    Number of params =         30

    --------------------------------------------------------------------------
         Factor  |     Variance   Difference        Proportion   Cumulative
```

```
    -------------+------------------------------------------------------------
        Factor1  |      3.01875      1.47811            0.5566       0.5566
        Factor2  |      1.54063      0.17267            0.2840       0.8406
        Factor3  |      1.36796            .            0.2522       1.0928
    --------------------------------------------------------------------------
    LR test: independent vs. saturated:  chi2(55) = 1012.53 Prob>chi2 = 0.0000

Rotated factor loadings (pattern matrix) and unique variances

    -----------------------------------------------------------
        Variable |  Factor1   Factor2   Factor3 |   Uniqueness 
    -------------+------------------------------+--------------
    pre1lassec~a |   0.3265              0.7109 |      0.3384  
    pre2elmalo~o |   0.2559              0.6762 |      0.4665  
    pre3lospro~n |   0.8181                     |      0.2486  
    pre4lospro~a |   0.8404                     |      0.2394  
    pre5molest~d |   0.5424              0.3412 |      0.5886  
    pre6losmar~e |   0.5336    0.3409           |      0.5500  
    pre7tinnit~í |   0.7293                     |      0.3959  
    pre8conqué~e |   0.2525    0.6369           |      0.4712  
    pre9realiz~s |   0.4680    0.4995           |      0.5196  
    pre10debid~e |             0.5910           |      0.6261  
    pre11conqu~e |   0.2654    0.5262           |      0.6284  
    -----------------------------------------------------------
    (blanks represent abs(loading)<.25)

Factor rotation matrix

    ---------------------------------------
                 | Factor1  Factor2  Factor3 
    -------------+--------------------------
         Factor1 |  0.7760   0.4469   0.4451 
         Factor2 | -0.5016   0.8651   0.0059 
         Factor3 | -0.3824  -0.2278   0.8955 
    ---------------------------------------

.
. rotate, promax horst blanks(.3)

Factor analysis/correlation                      Number of obs    =        200
    Method: principal factors                    Retained factors =          3
    Rotation: oblique promax (Kaiser on)         Number of params =         30

    --------------------------------------------------------------------------
         Factor  |     Variance   Proportion    Rotated factors are correlated
    -------------+------------------------------------------------------------
        Factor1  |      4.01959       0.7411
        Factor2  |      2.91809       0.5380
        Factor3  |      2.65472       0.4895
    --------------------------------------------------------------------------
    LR test: independent vs. saturated:  chi2(55) = 1012.53 Prob>chi2 = 0.0000

Rotated factor loadings (pattern matrix) and unique variances

    -----------------------------------------------------------
        Variable |  Factor1   Factor2   Factor3 |   Uniqueness 
    -------------+------------------------------+--------------
    pre1lassec~a |                       0.7102 |      0.3384  
    pre2elmalo~o |                       0.7121 |      0.4665  
    pre3lospro~n |   0.8467                     |      0.2486  
    pre4lospro~a |   0.8984                     |      0.2394  
    pre5molest~d |   0.5243                     |      0.5886  
    pre6losmar~e |   0.4567                     |      0.5500  
    pre7tinnit~í |   0.7486                     |      0.3959  
    pre8conqué~e |             0.6704           |      0.4712  
    pre9realiz~s |   0.3665    0.4803           |      0.5196  
    pre10debid~e |             0.6650           |      0.6261  
    pre11conqu~e |             0.5478           |      0.6284  
    -----------------------------------------------------------
    (blanks represent abs(loading)<.3)

Factor rotation matrix

    ---------------------------------------
                 | Factor1  Factor2  Factor3 
    -------------+--------------------------
         Factor1 |  0.9298   0.7532   0.7240 
         Factor2 | -0.3104   0.6465  -0.0139 
         Factor3 | -0.1977  -0.1211   0.6897 
    ---------------------------------------

.***Parallel Analysis

. fapara, reps(100)

PA -- Parallel Analysis for Factor Analysis -- N = 200
PA Eigenvalues Averaged Over 100 Replications
         FA         PA          Dif
  1.    4.532649    .4532064    4.079443
  2.    .8084863    .3329512    .4755351
  3.    .5862035    .2411771    .3450265
```

```
  4.    .1950031     .162653    .0323501
  5.    .0547553    .0955798   -.0408245
  6.   -.0289196    .0284292   -.0573488
  7.   -.0714914   -.0328738   -.0386176
  8.   -.1067984   -.0887762   -.0180223
  9.   -.1488288    -.152416    .0035871
 10.   -.1883742   -.2147534    .0263792
 11.   -.2088436   -.2761805    .0673369


.***THE ANALYSIS SHOWS CONFIRMS THE MODEL WITH THREE FACTORS AND THE 11 INITIAL QUESTIONS OF THE SCALE

.*******ANALYSIS WITH THE PARTITIONED SAMPLE

. set seed 512
.
. gen grupo = floor((2-1+1)*runiform() + 2)
.
. replace grupo=grupo-2
(200 real changes made)

. factortest pre1lassecrecionesoeldrena - pre11conquéfrecuenciaoque if grupo==0

Determinant of the correlation matrix
Det                =     0.018


Bartlett test of sphericity

Chi-square         =           379.063
Degrees of freedom =                55
p-value            =             0.000
H0: variables are not intercorrelated


Kaiser-Meyer-Olkin Measure of Sampling Adequacy
KMO               =     0.834


. polychoric pre1lassecrecionesoeldrena - pre11conquéfrecuenciaoque if grupo==0

Polychoric correlation matrix

                              pre1lassecrecionesoeldrena        pre2elmalolordeloído   pre3losproblemasparaoíren
 pre1lassecrecionesoeldrena                            1
      pre2elmalolordeloído                     .63882084                           1
 pre3losproblemasparaoíren                     .51176697                   .42220538                           1
pre4losproblemasparaoírcua                      .4089081                   .40236579                   .77516204
pre5molestiasdentroyalreded                     .44730226                   .32211293                   .45803287
 pre6losmareososensaciónde                     .39247615                   .29498669                   .55472498
 pre7tinnitusoruidoseneloí                     .40606689                   .42707886                    .6558389
  pre8conquéfrecuenciaoque                     .35939117                   .23042408                   .32183624
 pre9realizarsusactividades                     .48014346                   .48801319                   .55866965
  pre10debidoasuproblemaene                      .2000945                   .08040282                   .05074619
 pre11conquéfrecuenciaoque                      .3714107                   .26803619                   .43265184

                             pre4losproblemasparaoírcua  pre5molestiasdentroyalreded   pre6losmareososensaciónde
pre4losproblemasparaoírcua                            1
pre5molestiasdentroyalreded                     .45983489                           1
 pre6losmareososensaciónde                     .45111786                   .31052967                           1
 pre7tinnitusoruidoseneloí                     .59477907                   .45610356                    .5478254
  pre8conquéfrecuenciaoque                     .28985661                   .16074349                     .440085
 pre9realizarsusactividades                     .63060895                   .38114702                   .52925769
  pre10debidoasuproblemaene                    -.09270289                  -.06059961                   .26987992
 pre11conquéfrecuenciaoque                     .30404889                   .11511593                   .27748393

                              pre7tinnitusoruidoseneloí    pre8conquéfrecuenciaoque   pre9realizarsusactividades
 pre7tinnitusoruidoseneloí                            1
  pre8conquéfrecuenciaoque                     .22111049                           1
 pre9realizarsusactividades                     .52181502                   .49096349                           1
  pre10debidoasuproblemaene                    -.02614323                   .49507833                   .13019425
 pre11conquéfrecuenciaoque                     .29464829                   .38636611                   .44151561

                               pre10debidoasuproblemaene   pre11conquéfrecuenciaoque
  pre10debidoasuproblemaene                            1
 pre11conquéfrecuenciaoque                     .29674857                           1

.
. matrix poly2=r(R)

. *Analisis factorial sin la pregunta 12 (con base reducida)

.
.
.
. factormat poly2, n(100) factor(3)
(obs=100)

Factor analysis/correlation                      Number of obs    =        100
    Method: principal factors                    Retained factors =          3
```

```
    Rotation: (unrotated)                        Number of params =         30

    --------------------------------------------------------------------------
         Factor  |   Eigenvalue   Difference        Proportion   Cumulative
    -------------+------------------------------------------------------------
        Factor1  |      4.47471      3.42660            0.7951       0.7951
        Factor2  |      1.04812      0.58496            0.1862       0.9814
        Factor3  |      0.46316      0.26521            0.0823       1.0637
        Factor4  |      0.19795      0.07657            0.0352       1.0988
        Factor5  |      0.12138      0.06178            0.0216       1.1204
        Factor6  |      0.05960      0.08747            0.0106       1.1310
        Factor7  |     -0.02787      0.08823           -0.0050       1.1260
        Factor8  |     -0.11610      0.05135           -0.0206       1.1054
        Factor9  |     -0.16746      0.01621           -0.0298       1.0757
       Factor10  |     -0.18367      0.05845           -0.0326       1.0430
       Factor11  |     -0.24212            .           -0.0430       1.0000
    --------------------------------------------------------------------------
    LR test: independent vs. saturated:  chi2(55) =  507.93 Prob>chi2 = 0.0000

Factor loadings (pattern matrix) and unique variances

    -----------------------------------------------------------
        Variable |  Factor1   Factor2   Factor3 |   Uniqueness
    -------------+------------------------------+--------------
    pre1lassec~a |   0.6851    0.0847    0.3992 |      0.3640
    pre2elmalo~o |   0.6026   -0.0366    0.4115 |      0.4662
    pre3lospro~n |   0.8260   -0.1885   -0.1634 |      0.2555
    pre4lospro~a |   0.7668   -0.3161   -0.1841 |      0.2783
    pre5molest~d |   0.5292   -0.2501    0.1073 |      0.6459
    pre6losmar~e |   0.6608    0.1359   -0.2002 |      0.5048
    pre7tinnit~í |   0.7117   -0.2418   -0.0925 |      0.4265
    pre8conqué~e |   0.5165    0.5071   -0.1039 |      0.4653
    pre9realiz~s |   0.7718    0.0619   -0.0422 |      0.3987
    pre10debid~e |   0.1925    0.6503   -0.0196 |      0.5397
    pre11conqu~e |   0.4992    0.2842   -0.0282 |      0.6693
    -----------------------------------------------------------

. rotate, factor(3) blank(0.25) varimax

Factor analysis/correlation                      Number of obs    =        100
    Method: principal factors                    Retained factors =          3
    Rotation: orthogonal varimax (Kaiser off)    Number of params =         30

    --------------------------------------------------------------------------
         Factor  |     Variance   Difference        Proportion   Cumulative
    -------------+------------------------------------------------------------
        Factor1  |      3.21243      1.77763            0.5708       0.5708
        Factor2  |      1.43480      0.09603            0.2550       0.8258
        Factor3  |      1.33876            .            0.2379       1.0637
    --------------------------------------------------------------------------
    LR test: independent vs. saturated:  chi2(55) =  507.93 Prob>chi2 = 0.0000

Rotated factor loadings (pattern matrix) and unique variances

    -----------------------------------------------------------
        Variable |  Factor1   Factor2   Factor3 |   Uniqueness
    -------------+------------------------------+--------------
    pre1lassec~a |   0.3558              0.6749 |      0.3640
    pre2elmalo~o |   0.3325              0.6443 |      0.4662
    pre3lospro~n |   0.8164                     |      0.2555
    pre4lospro~a |   0.8284                     |      0.2783
    pre5molest~d |   0.4864              0.3361 |      0.6459
    pre6losmar~e |   0.5666    0.3949           |      0.5048
    pre7tinnit~í |   0.7152                     |      0.4265
    pre8conqué~e |   0.2591    0.6645           |      0.4653
    pre9realiz~s |   0.6205    0.3330    0.3247 |      0.3987
    pre10debid~e |             0.6657           |      0.5397
    pre11conqu~e |   0.3031    0.4389           |      0.6693
    -----------------------------------------------------------
    (blanks represent abs(loading)<.25)

Factor rotation matrix

    -----------------------------------------
                 | Factor1  Factor2  Factor3
    -------------+---------------------------
         Factor1 |  0.8134   0.3467   0.4671
         Factor2 | -0.4034   0.9147   0.0236
         Factor3 | -0.4191  -0.2076   0.8839
    -----------------------------------------

.
. rotate, promax horst blanks(.3)

Factor analysis/correlation                      Number of obs    =        100
    Method: principal factors                    Retained factors =          3
    Rotation: oblique promax (Kaiser on)         Number of params =         30

    --------------------------------------------------------------------------
         Factor  |     Variance   Proportion    Rotated factors are correlated
```

```
    -------------+------------------------------------------------------------
        Factor1  |      4.05402       0.7204
        Factor2  |      2.91694       0.5183
        Factor3  |      2.18075       0.3875
    --------------------------------------------------------------------------
    LR test: independent vs. saturated:  chi2(55) =  507.93 Prob>chi2 = 0.0000

Rotated factor loadings (pattern matrix) and unique variances

    -----------------------------------------------------------
        Variable |  Factor1   Factor2   Factor3 |   Uniqueness 
    -------------+------------------------------+--------------
    pre1lassec~a |             0.6883           |      0.3640  
    pre2elmalo~o |             0.6854           |      0.4662  
    pre3lospro~n |   0.8309                     |      0.2555  
    pre4lospro~a |   0.8850                     |      0.2783  
    pre5molest~d |   0.4255                     |      0.6459  
    pre6losmar~e |   0.5520              0.3465 |      0.5048  
    pre7tinnit~í |   0.7218                     |      0.4265  
    pre8conqué~e |                       0.6719 |      0.4653  
    pre9realiz~s |   0.5359                     |      0.3987  
    pre10debid~e |                       0.7236 |      0.5397  
    pre11conqu~e |                       0.4155 |      0.6693  
    -----------------------------------------------------------
    (blanks represent abs(loading)<.3)

Factor rotation matrix

    -----------------------------------------
                 | Factor1  Factor2  Factor3 
    -------------+---------------------------
         Factor1 |  0.9408   0.7821   0.5765 
         Factor2 | -0.2141   0.6232  -0.1028 
         Factor3 | -0.2629  -0.0009   0.8106 
    -----------------------------------------

.
. matrix factor2=e(r_L)

. **Adicionales
.
. *Comunalidad
.
. estat smc

Squared multiple correlations of variables with all other variables

    -----------------------
        Variable |     smc 
    -------------+---------
    pre1lassec~a |  0.5537 
    pre2elmalo~o |  0.4769 
    pre3lospro~n |  0.7239 
    pre4lospro~a |  0.6952 
    pre5molest~d |  0.3472 
    pre6losmar~e |  0.4926 
    pre7tinnit~í |  0.5464 
    pre8conqué~e |  0.4604 
    pre9realiz~s |  0.6009 
    pre10debid~e |  0.3787 
    pre11conqu~e |  0.3519 
    -----------------------

.
. *Factores Bondad de ajuste

.
. estat factors, factors(3)

Factor analysis with different numbers of factors (maximum likelihood)

    ----------------------------------------------------------
    #factors |     loglik   df_m   df_r        AIC        BIC
    ---------+------------------------------------------------
           1 |  -72.72784     11     44   167.4557   196.1126
           2 |  -37.78287     21     34   117.5657   172.2743
           3 |  -20.93227     30     25   101.8645   180.0196
    ----------------------------------------------------------
    the model with 3 factors is a Heywood case
.
. *Matriz de correlacion
.
. estat common

Correlation matrix of the promax(3) rotated common factors

    --------------------------------------------
         Factors |  Factor1   Factor2   Factor3 
    -------------+------------------------------
         Factor1 |        1 
         Factor2 |    .6026         1 
         Factor3 |    .3512     .3861         1 
```

```
    ------------------------------------------
.
. tab grupo

      grupo |      Freq.     Percent        Cum.
------------+-----------------------------------
          0 |        100       50.00       50.00
          1 |        100       50.00      100.00
------------+-----------------------------------
      Total |        200      100.00

. keep if grupo==1
(100 observations deleted)

. tab grupo

      grupo |      Freq.     Percent        Cum.
------------+-----------------------------------
          1 |        100      100.00      100.00
------------+-----------------------------------
      Total |        100      100.00

. sem (Secrecionymalolor -> pre1lassecrecionesoeldrena, ) (Secrecionymalolor -> pre2elmalolordeloído, ) (Problemasauditivos ->
> pre3losproblemasparaoíren, ) (Problemasauditivos -> pre4losproblemasparaoírcua, ) (Problemasauditivos -> pre5molestiasdentroy
> alreded, ) (Problemasauditivos -> pre6losmareosοsensaciónde, ) (Problemasauditivos -> pre7tinnitusoruidoseneloí, ) (Impactosa
> ludestilovida -> pre8conquéfrecuenciaoque, ) (Impactosaludestilovida -> pre9realizarsusactividades, ) (Impactosaludestilovida
>  -> pre10debidoasuproblemaene, ) (Impactosaludestilovida -> pre11conquéfrecuenciaoque, ), covstruct(_lexogenous, diagonal) la
> tent(Secrecionymalolor Problemasauditivos Impactosaludestilovida ) cov( Secrecionymalolor*Problemasauditivos Problemasauditiv
> os*Impactosaludestilovida Impactosaludestilovida*Secrecionymalolor) nocapslatent

Endogenous variables

Measurement:  pre1lassecrecionesoeldrena pre2elmalolordeloído pre3losproblemasparaoíren pre4losproblemasparaoírcua
              pre5molestiasdentroyalreded pre6losmareosοsensaciónde pre7tinnitusoruidoseneloí pre8conquéfrecuenciaoque
              pre9realizarsusactividades pre10debidoasuproblemaene pre11conquéfrecuenciaoque

Exogenous variables

Latent:       Secrecionymalolor Problemasauditivos Impactosaludestilovida

Fitting target model:

Iteration 0:   log likelihood = -1855.1005
Iteration 1:   log likelihood = -1853.8304
Iteration 2:   log likelihood = -1853.4373
Iteration 3:   log likelihood = -1853.1282
Iteration 4:   log likelihood =  -1853.112  (backed up)
Iteration 5:   log likelihood =  -1853.108  (backed up)
Iteration 6:   log likelihood = -1853.1078  (backed up)
Iteration 7:   log likelihood = -1853.1077  (backed up)
Iteration 8:   log likelihood = -1853.1076  (backed up)
Iteration 9:   log likelihood = -1853.1076  (backed up)
Iteration 10:  log likelihood = -1853.1076  (backed up)
Iteration 11:  log likelihood = -1853.1076  (backed up)
Iteration 12:  log likelihood = -1853.1076  (backed up)
Iteration 13:  log likelihood = -1853.1076  (backed up)
Iteration 14:  log likelihood = -1853.1076  (backed up)
Iteration 15:  log likelihood = -1853.1076  (backed up)
Iteration 16:  log likelihood = -1853.1076  (backed up)
Iteration 17:  log likelihood = -1853.1076  (backed up)
Iteration 18:  log likelihood = -1853.1076  (backed up)
Iteration 19:  log likelihood = -1853.1076  (backed up)
Iteration 20:  log likelihood = -1853.1076  (backed up)
Iteration 21:  log likelihood = -1853.1076  (backed up)
Iteration 22:  log likelihood = -1853.1076  (backed up)
Iteration 23:  log likelihood = -1853.1076  (backed up)
Iteration 24:  log likelihood = -1853.1076  (backed up)
Iteration 25:  log likelihood = -1853.1076  (backed up)
Iteration 26:  log likelihood = -1853.1076  (backed up)
Iteration 27:  log likelihood = -1853.1076  (backed up)
Iteration 28:  log likelihood = -1853.1076  (backed up)
Iteration 29:  log likelihood = -1853.1076  (backed up)
Iteration 30:  log likelihood = -1853.1076  (backed up)
Iteration 31:  log likelihood = -1853.1076  (backed up)
Iteration 32:  log likelihood = -1853.1076  (backed up)
Iteration 33:  log likelihood = -1853.1076  (backed up)
Iteration 34:  log likelihood = -1853.1076  (backed up)
Iteration 35:  log likelihood = -1853.1076  (backed up)
Iteration 36:  log likelihood = -1853.1076  (backed up)
Iteration 37:  log likelihood = -1853.1076  (backed up)
Iteration 38:  log likelihood = -1853.1076  (backed up)
Iteration 39:  log likelihood = -1853.1076  (backed up)
Iteration 40:  log likelihood = -1853.1076  (backed up)
Iteration 41:  log likelihood = -1853.1076  (backed up)
Iteration 42:  log likelihood = -1853.1076  (backed up)
Iteration 43:  log likelihood = -1853.1076  (backed up)
Iteration 44:  log likelihood = -1853.1076  (backed up)
Iteration 45:  log likelihood = -1853.1076  (backed up)
Iteration 46:  log likelihood = -1853.1076  (backed up)
Iteration 47:  log likelihood = -1853.1076  (backed up)
```

```
Iteration 48:  log likelihood = -1853.1076  (backed up)
Iteration 49:  log likelihood = -1853.1076  (backed up)
Iteration 50:  log likelihood = -1853.1076  (backed up)
Iteration 51:  log likelihood = -1853.1076  (backed up)
Iteration 52:  log likelihood = -1853.1076  (backed up)
Iteration 53:  log likelihood = -1853.1076  (backed up)
Iteration 54:  log likelihood = -1853.1076  (backed up)
Iteration 55:  log likelihood = -1853.1076  (backed up)
Iteration 56:  log likelihood = -1853.1076  (backed up)
Iteration 57:  log likelihood = -1853.1076  (backed up)
Iteration 58:  log likelihood = -1853.1076  (backed up)
Iteration 59:  log likelihood = -1853.1076  (backed up)
Iteration 60:  log likelihood = -1853.1076  (backed up)
Iteration 61:  log likelihood = -1853.1076  (backed up)
Iteration 62:  log likelihood = -1853.1076  (backed up)
Iteration 63:  log likelihood = -1853.1076  (backed up)
Iteration 64:  log likelihood = -1853.1076  (backed up)
Iteration 65:  log likelihood = -1853.1076  (backed up)
Iteration 66:  log likelihood = -1853.1076  (backed up)
Iteration 67:  log likelihood = -1853.1076  (backed up)
Iteration 68:  log likelihood = -1853.1076  (backed up)
Iteration 69:  log likelihood = -1853.1076  (backed up)
Iteration 70:  log likelihood = -1853.1076  (backed up)
Iteration 71:  log likelihood = -1853.1076  (backed up)
Iteration 72:  log likelihood = -1853.1076  (backed up)
Iteration 73:  log likelihood = -1853.1076  (backed up)
Iteration 74:  log likelihood = -1853.1076  (backed up)
Iteration 75:  log likelihood = -1853.1076  (backed up)
Iteration 76:  log likelihood = -1853.1076  (backed up)
Iteration 77:  log likelihood = -1853.1076  (backed up)
Iteration 78:  log likelihood = -1853.1076  (backed up)
Iteration 79:  log likelihood = -1853.1076  (backed up)
Iteration 80:  log likelihood = -1853.1076  (backed up)
Iteration 81:  log likelihood = -1853.1076  (backed up)
Iteration 82:  log likelihood = -1853.1076  (backed up)
Iteration 83:  log likelihood = -1853.1076  (backed up)
Iteration 84:  log likelihood = -1853.1076  (backed up)
Iteration 85:  log likelihood = -1853.1076  (backed up)
Iteration 86:  log likelihood = -1853.1076  (backed up)
Iteration 87:  log likelihood = -1853.1076  (backed up)
Iteration 88:  log likelihood = -1853.1076  (backed up)
Iteration 89:  log likelihood = -1853.1076  (backed up)
Iteration 90:  log likelihood = -1853.1076  (backed up)
Iteration 91:  log likelihood = -1853.1076  (backed up)
Iteration 92:  log likelihood = -1853.1076  (backed up)
Iteration 93:  log likelihood = -1853.1076  (backed up)
Iteration 94:  log likelihood = -1853.1076  (backed up)
Iteration 95:  log likelihood = -1853.1076  (backed up)
Iteration 96:  log likelihood = -1853.1076  (backed up)
Iteration 97:  log likelihood = -1853.1076  (backed up)
Iteration 98:  log likelihood = -1853.1076  (backed up)
Iteration 99:  log likelihood = -1853.1076  (backed up)
Iteration 100: log likelihood = -1853.1076  (backed up)
Iteration 101: log likelihood = -1853.1076  (backed up)
Iteration 102: log likelihood = -1853.1076  (backed up)
Iteration 103: log likelihood = -1853.1076  (backed up)
Iteration 104: log likelihood = -1853.1076  (backed up)
Iteration 105: log likelihood = -1853.1076  (backed up)
Iteration 106: log likelihood = -1853.1076  (backed up)
Iteration 107: log likelihood = -1853.1076  (backed up)
Iteration 108: log likelihood = -1853.1076  (backed up)
Iteration 109: log likelihood = -1853.1076  (backed up)
Iteration 110: log likelihood = -1853.1076  (backed up)
Iteration 111: log likelihood = -1853.1076  (backed up)
Iteration 112: log likelihood = -1853.1076  (backed up)
Iteration 113: log likelihood = -1853.1076  (backed up)
Iteration 114: log likelihood = -1853.1076  (backed up)
Iteration 115: log likelihood = -1853.1076  (backed up)
Iteration 116: log likelihood = -1853.1076  (backed up)
Iteration 117: log likelihood = -1853.1076  (backed up)
Iteration 118: log likelihood = -1853.1076  (backed up)
Iteration 119: log likelihood = -1853.1076  (backed up)
Iteration 120: log likelihood = -1853.1076  (backed up)
Iteration 121: log likelihood = -1853.1076  (backed up)
Iteration 122: log likelihood = -1853.1076  (backed up)
Iteration 123: log likelihood = -1853.1076  (backed up)
Iteration 124: log likelihood = -1853.1076  (backed up)
Iteration 125: log likelihood = -1853.1076  (backed up)
Iteration 126: log likelihood = -1853.1076  (backed up)
Iteration 127: log likelihood = -1853.1076  (backed up)
Iteration 128: log likelihood = -1853.1076  (backed up)
Iteration 129: log likelihood = -1853.1076  (backed up)
Iteration 130: log likelihood = -1853.1076  (backed up)
Iteration 131: log likelihood = -1853.1076  (backed up)
Iteration 132: log likelihood = -1853.1076  (backed up)
Iteration 133: log likelihood = -1853.1076  (backed up)
Iteration 134: log likelihood = -1853.1076  (backed up)
Iteration 135: log likelihood = -1853.1076  (backed up)
Iteration 136: log likelihood = -1853.1076  (backed up)
Iteration 137: log likelihood = -1853.1076  (backed up)
```

```
Iteration 138: log likelihood = -1853.1076  (backed up)
Iteration 139: log likelihood = -1853.1076  (backed up)
Iteration 140: log likelihood = -1853.1076  (backed up)
Iteration 141: log likelihood = -1853.1076  (backed up)
Iteration 142: log likelihood = -1853.1076  (backed up)
Iteration 143: log likelihood = -1853.1076  (backed up)
Iteration 144: log likelihood = -1853.1076  (backed up)
Iteration 145: log likelihood = -1853.1076  (backed up)
Iteration 146: log likelihood = -1853.1076  (backed up)
Iteration 147: log likelihood = -1853.1076  (backed up)
Iteration 148: log likelihood = -1853.1076  (backed up)
Iteration 149: log likelihood = -1853.1076  (backed up)
Iteration 150: log likelihood = -1853.1076  (backed up)
Iteration 151: log likelihood = -1853.1076  (backed up)
Iteration 152: log likelihood = -1853.1076  (backed up)
Iteration 153: log likelihood = -1853.1076  (backed up)
Iteration 154: log likelihood = -1853.1076  (backed up)
Iteration 155: log likelihood = -1853.1076  (backed up)
Iteration 156: log likelihood = -1853.1076  (backed up)
Iteration 157: log likelihood = -1853.1076  (backed up)
Iteration 158: log likelihood = -1853.1076  (backed up)
Iteration 159: log likelihood = -1853.1076  (backed up)
Iteration 160: log likelihood = -1853.1076  (backed up)
Iteration 161: log likelihood = -1853.1076  (backed up)
Iteration 162: log likelihood = -1853.1076  (backed up)
Iteration 163: log likelihood = -1853.1076  (backed up)
Iteration 164: log likelihood = -1853.1076  (backed up)
Iteration 165: log likelihood = -1853.1076  (backed up)
Iteration 166: log likelihood = -1853.1076  (backed up)
Iteration 167: log likelihood = -1853.1076  (backed up)
Iteration 168: log likelihood = -1853.1076  (backed up)
Iteration 169: log likelihood = -1853.1076  (backed up)
Iteration 170: log likelihood = -1853.1076  (backed up)
Iteration 171: log likelihood = -1853.1076  (backed up)
Iteration 172: log likelihood = -1853.1076  (backed up)
Iteration 173: log likelihood = -1853.1076  (backed up)
Iteration 174: log likelihood = -1853.1076  (backed up)
Iteration 175: log likelihood = -1853.1076  (backed up)
Iteration 176: log likelihood = -1853.1076  (backed up)
Iteration 177: log likelihood = -1853.1076  (backed up)
Iteration 178: log likelihood = -1853.1076  (backed up)
Iteration 179: log likelihood = -1853.1076  (backed up)
Iteration 180: log likelihood = -1853.1076  (backed up)
Iteration 181: log likelihood = -1853.1076  (backed up)
Iteration 182: log likelihood = -1853.1076  (backed up)
Iteration 183: log likelihood = -1853.1076  (backed up)
Iteration 184: log likelihood = -1853.1076  (backed up)
Iteration 185: log likelihood = -1853.1076  (backed up)
Iteration 186: log likelihood = -1853.1076  (backed up)
Iteration 187: log likelihood = -1853.1076  (backed up)
Iteration 188: log likelihood = -1853.1076  (backed up)
Iteration 189: log likelihood = -1853.1076  (backed up)
Iteration 190: log likelihood = -1853.1076  (backed up)
Iteration 191: log likelihood = -1853.1076  (backed up)
Iteration 192: log likelihood = -1853.1076  (backed up)
Iteration 193: log likelihood = -1853.1076  (backed up)
Iteration 194: log likelihood = -1853.1076  (backed up)
Iteration 195: log likelihood = -1853.1076  (backed up)
Iteration 196: log likelihood = -1853.1076  (backed up)
Iteration 197: log likelihood = -1853.1076  (backed up)
Iteration 198: log likelihood = -1853.1076  (backed up)
Iteration 199: log likelihood = -1853.1076  (backed up)
Iteration 200: log likelihood = -1853.1076  (backed up)
convergence not achieved

Structural equation model                       Number of obs     =        100
Estimation method  = ml
Log likelihood     = -1853.1076

 ( 1)  [pre1lassecrecionesoeldrena]Secrecionymalolor = 1
 ( 2)  [pre3losproblemasparaoíren]Problemasauditivos = 1
 ( 3)  [pre8conquéfrecuenciaoque]Impactosaludestilovida = 1
------------------------------------------------------------------------------------------------------
                                               |                 OIM
                                               |      Coef.   Std. Err.      z    P>|z|     [95% Conf. Interval]
-----------------------------------------------+------------------------------------------------------
Measurement                                    |
  pre1lassecrecionesoeldrena                   |
                           Secrecionymalolor |          1  (constrained)
                                       _cons |       3.29    .1465932    22.44   0.000     3.002683    3.577317
  ---------------------------------------------+------------------------------------------------------
  pre2elmalolordeloído                         |
                           Secrecionymalolor |    .742051    .1640451     4.52   0.000     .4205285    1.063574
                                       _cons |       3.09    .1849578    16.71   0.000     2.727489    3.452511
  ---------------------------------------------+------------------------------------------------------
  pre3losproblemasparaoíren                    |
                          Problemasauditivos |          1  (constrained)
                                       _cons |       3.94     .128199    30.73   0.000     3.688735    4.191265
  ---------------------------------------------+------------------------------------------------------
  pre4losproblemasparaoírcua                   |
```

```
                        Problemasauditivos |   .9259157   .0830166    11.15   0.000     .7632062    1.088625
                                     _cons |       3.97   .1193837    33.25   0.000     3.736012    4.203988
  -----------------------------------------+----------------------------------------------------------------
  pre5molestiasdentroyalreded              |
                        Problemasauditivos |   .9112768   .1373528     6.63   0.000     .6420702    1.180483
                                     _cons |       3.33   .1654889    20.12   0.000     3.005648    3.654352
  -----------------------------------------+----------------------------------------------------------------
  pre6losmareososensaciónde                |
                        Problemasauditivos |   .8518748    .158591     5.37   0.000     .5410422    1.162707
                                     _cons |       2.64   .1813413    14.56   0.000     2.284578    2.995422
  -----------------------------------------+----------------------------------------------------------------
  pre7tinnitusoruidosenelоí                |
                        Problemasauditivos |   1.082396    .126303     8.57   0.000     .8348471    1.329946
                                     _cons |       3.61      .1623    22.24   0.000     3.291898    3.928102
  -----------------------------------------+----------------------------------------------------------------
  pre8conquéfrecuenciaoque                 |
                    Impactosaludestilovida |          1  (constrained)
                                     _cons |       2.41   .1837167    13.12   0.000     2.049922    2.770078
  -----------------------------------------+----------------------------------------------------------------
  pre9realizarsusactividades               |
                    Impactosaludestilovida |   .6591434   .1445589     4.56   0.000     .3758132    .9424736
                                     _cons |       2.98   .1725172    17.27   0.000     2.641873    3.318127
  -----------------------------------------+----------------------------------------------------------------
  pre10debidoasuproblemaene                |
                    Impactosaludestilovida |   .5342918    .139178     3.84   0.000     .2615079    .8070757
                                     _cons |       4.24     .16737    25.33   0.000     3.911961    4.568039
  -----------------------------------------+----------------------------------------------------------------
  pre11conquéfrecuenciaoque                |
                    Impactosaludestilovida |    .507158   .0945417     5.36   0.000     .3218597    .6924564
                                     _cons |       3.02   .1221846    24.72   0.000     2.780523    3.259477
-------------------------------------------+----------------------------------------------------------------
           var(e.pre1lassecrecionesoeldrena)|  -1.00e-09   .4045776                             .           .
                 var(e.pre2elmalolordeloído)|    2.23764   .3851168                      1.596949    3.135373
            var(e.pre3losproblemasparaoíren)|   .3809856   .0753481                      .2585623    .5613736
           var(e.pre4losproblemasparaoírcua)|   .3428688   .0707628                      .2287985    .5138103
          var(e.pre5molestiasdentroyalreded)|   1.690234   .2557145                      1.256519    2.273655
            var(e.pre6losmareososensaciónde)|   2.372272    .350767                      1.775435    3.169744
            var(e.pre7tinnitusoruidosenelоí)|   1.154992   .1869275                      .8410425    1.586135
             var(e.pre8conquéfrecuenciaoque)|   1.224601   .3320231                      .7197975    2.083429
           var(e.pre9realizarsusactividades)|   2.041856   .3449438                      1.466311    2.843308
            var(e.pre10debidoasuproblemaene)|    2.18735   .3354564                      1.619482    2.954338
            var(e.pre11conquéfrecuenciaoque)|   .9397573   .1680468                      .6619183    1.334219
                      var(Secrecionymalolor)|   2.148957   .5007547                      1.361065    3.392943
                     var(Problemasauditivos)|   1.262513   .2376521                      .8729874    1.825844
                 var(Impactosaludestilovida)|    2.15058   .5208454                      1.337841    3.457058
-------------------------------------------+----------------------------------------------------------------
     cov(Secrecionymalolor,Problemasauditivos)|   .8679783   .2073019     4.19   0.000     .4616742    1.274283
 cov(Secrecionymalolor,Impactosaludestilovida)|   1.125302   .2978059     3.78   0.000     .5416134    1.708991
cov(Problemasauditivos,Impactosaludestilovida)|   1.057588   .2446189     4.32   0.000     .5781436    1.537032
------------------------------------------------------------------------------------------------------------
LR test of model vs. saturated: chi2(41)  =     62.75, Prob > chi2 = 0.0160
convergence not achieved
r(430);

.
. estimate store modelo3factred

.
. estat gof, stats(all)

----------------------------------------------------------------------------
Fit statistic        |      Value   Description
---------------------+------------------------------------------------------
Likelihood ratio     |
         chi2_ms(41) |     62.753   model vs. saturated
            p > chi2 |      0.016
         chi2_bs(55) |    451.473   baseline vs. saturated
            p > chi2 |      0.000
---------------------+------------------------------------------------------
Population error     |
               RMSEA |      0.073   Root mean squared error of approximation
 90% CI, lower bound |      0.032
         upper bound |      0.108
              pclose |      0.147   Probability RMSEA <= 0.05
---------------------+------------------------------------------------------
Information criteria |
                 AIC |   3778.215   Akaike's information criterion
                 BIC |   3872.001   Bayesian information criterion
---------------------+------------------------------------------------------
Baseline comparison  |
                 CFI |      0.945   Comparative fit index
                 TLI |      0.926   Tucker-Lewis index
---------------------+------------------------------------------------------
Size of residuals    |
                SRMR |      0.063   Standardized root mean squared residual
                  CD |      1.000   Coefficient of determination
----------------------------------------------------------------------------

. log close
      name:  <unnamed>
```

```
      log:  C:\Users\smmor\Desktop\Analisis factorial adicional COMQ-12 03 jul 2020.log
  log type:  text
 closed on:   3 Jul 2020, 20:42:15
-----------------------------------------------------------------------------------------------------------------------------
```
